# Diatoms can be an important exception to temperature–size rules at species and community levels of organization

**DOI:** 10.1111/gcb.12285

**Published:** 2013-08-18

**Authors:** Georgina L Adams, Doris E Pichler, Eileen J Cox, Eoin J O'Gorman, Alex Seeney, Guy Woodward, Daniel C Reuman

**Affiliations:** * Imperial College London, Silwood Park CampusBuckhurst Road, Ascot, Berkshire, SL5 7PY, UK; † School of Biological and Chemical Sciences, Queen Mary University of LondonMile End Road, London, E1 4NS, UK; ‡ Natural History MuseumCromwell Road, London, SW7 5BD, UK; § Laboratory of Populations, Rockefeller University1230 York Ave, New York, NY, 10065, USA

**Keywords:** Bergmann's rule, climate change, community size structure, diatoms, global warming, James’ rule, phytobenthos, phytoplankton, temperature–size relationships, temperature–size rule

## Abstract

Climate warming has been linked to an apparent general decrease in body sizes of ectotherms, both across and within taxa, especially in aquatic systems. Smaller body size in warmer geographical regions has also been widely observed. Since body size is a fundamental determinant of many biological attributes, climate-warming-related changes in size could ripple across multiple levels of ecological organization. Some recent studies have questioned the ubiquity of temperature–size rules, however, and certain widespread and abundant taxa, such as diatoms, may be important exceptions. We tested the hypothesis that diatoms are smaller at warmer temperatures using a system of geothermally heated streams. There was no consistent relationship between size and temperature at either the population or community level. These field data provide important counterexamples to both James’ and Bergmann's temperature–size rules, respectively, undermining the widely held assumption that warming favours the small. This study provides compelling new evidence that diatoms are an important exception to temperature–size rules for three reasons: (i) we use many more species than prior work; (ii) we examine both community and species levels of organization simultaneously; (iii) we work in a natural system with a wide temperature gradient but minimal variation in other factors, to achieve robust tests of hypotheses without relying on laboratory setups, which have limited realism. In addition, we show that interspecific effects were a bigger contributor to whole-community size differences, and are probably more ecologically important than more commonly studied intraspecific effects. These findings highlight the need for multispecies approaches in future studies of climate warming and body size.

## Introduction

Body size is a key determinant of organism physiological traits and functional roles within an ecosystem (Peters 1983; Cohen *et al*. 2003; Brown *et al*. 2004; Woodward *et al*. 2005). Numerous studies have linked temperature and climate warming with changes in body size across taxa, with an apparent trend that warmer conditions tend to favour smaller organisms (Atkinson 1994; Daufresne *et al*. 2009; Sheridan & Bickford 2011; Yom-Tov *et al*. 2010; Gardner *et al*. 2009; Tarone *et al*. 2011; Stelzer 2002; Atkinson *et al*. 2003). Examples of larger or unchanged sizes at warmer temperatures also exist, but these are far less commonly reported (Sheridan & Bickford 2011). Temperature–size relationships have ramifications for ecological interactions and the structure and functioning of communities, such that warming-induced changes in organism size and its consequences have been called one of the most far-reaching consequences of climate change (Daufresne *et al*. 2009).

The general trend of smaller sizes at warmer temperatures has been separated conceptually into three well-known relationships, which are related to each other but are distinct in detail: Bergmann's rule (Bergmann 1847), James’ rule (James 1970), and the temperature–size rule (Atkinson 1994, 1995). Bergmann's rule states that larger species tend to be found at higher latitudes, and therefore at colder temperatures (Blackburn *et al*. 1999; Angilletta *et al*. 2004; Millien *et al*. 2006; Georg & Christian 2011). James’ rule is an intraspecific version of Bergmann's rule (Blackburn *et al*. 1999) and states that the mean body size of a species will be smaller at warmer temperatures. The temperature–size rule states that size at a fixed age or developmental stage is smaller in warmer temperatures for ectotherms (Atkinson 1994; Atkinson & Sibly 1997), and results from a plastic response at the individual level. This study focuses on Bergmann's and James’ rules.

Recent work suggests that, for ectotherms in aquatic environments, the trend of smaller sizes at warmer temperatures is sufficiently widespread, in both experimental and survey data, that it may be a universal rule (Daufresne *et al*. 2009). Daufresne *et al*. (2009) also suggested that “… a common mechanism (or set of mechanisms) links size structure and thermal energy at all biological scales” (p. 12790) and speculated that the metabolic theory of ecology (Brown *et al*. 2004) could be implicated in such an explanation, although they did not explore or test possible mechanisms in detail. Crucially, this depends on temperature–body size effects at those levels being in agreement. The analyses of Daufresne *et al*. (2009) are far-reaching and compelling, but the true universality and the underlying mechanisms behind the effects they describe require further research. Such potential mechanisms may be illuminated as much by exceptions as to any agreements with general trends.

Diatoms represent an ideal group of model organisms to test these ideas, as past research on warming-related body-size changes has produced mixed results. We use the term body size here in place of cell size, since it is understood in its broader sense in the application of the temperature–size rules. Diatoms are found in almost all aquatic systems (Smol & Stoermer 2010) and account for around 23% of the world's total primary production (Snoeijs *et al*. 2002). They play a key role in aquatic food web structure because their cell size can influence the flow of energy to higher trophic levels, as well as influencing an ecosystem's carbon cycle (Smetacek 1999; Yvon-Durocher *et al*. 2010; O'Gorman *et al*. 2012). There is equivocal evidence that diatoms follow the temperature–size rule in controlled temperature studies at the intraspecific level (Montagnes & Franklin 2001; Atkinson *et al*. 2003). At the community level, shifts have been observed towards smaller primary producer species at warmer temperatures in both mesocosm experiments (Lewandowska & Sommer 2010; Yvon-Durocher *et al*. 2011; Peter & Sommer 2012) and natural ecosystems (Li *et al*. 2009; Winder *et al*. 2009; Barnes *et al*. 2010), as predicted by Daufresne *et al*. (2009). However, exceptions have also been found (Atkinson 1995; O'Gorman *et al*. 2012; Rüger & Sommer 2012) and if diatoms (and other single-celled organisms) do not conform to the rule of smaller body sizes at warmer temperatures, then they are very important exceptions. The first and main goal of this article is to determine to what extent diatoms exhibit decreases in body size at the community level (Bergmann's rule) and intraspecifically (James’ rule), under warming.

**Figure 1:**
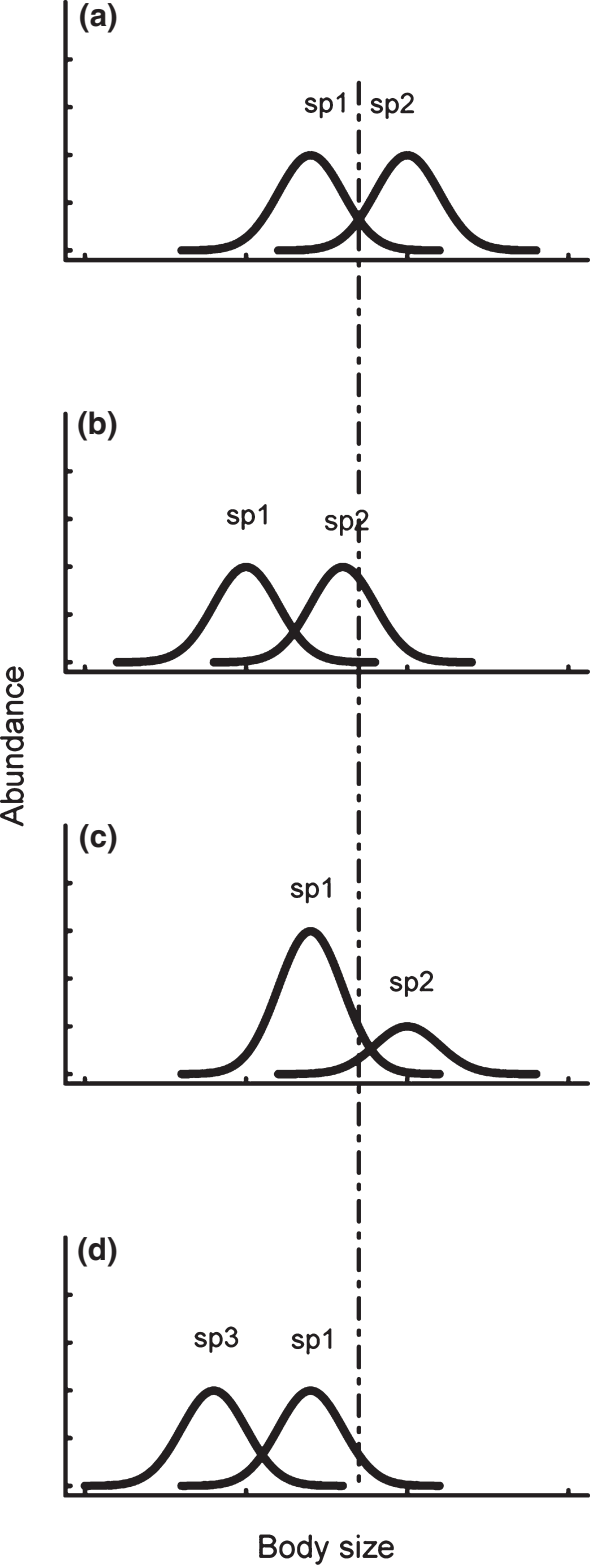
Plots showing conceptual differences among possible causes of overall community size changes with warming, for a two-species community. Compared to the reference distribution (a), community mean size could decrease from either the mean size of individual species decreasing (b), the relative abundance of small species increasing (c), species turnover effects (d) or a combination. The dashed line shows the mean body size in the reference distribution (a), and is for reference. Changes towards larger sizes could occur as well via the same three distinct routes or a combination. The abbreviation ‘sp’ is for ‘species’.

It is critically important to understand how climate change affects whole assemblages or communities, yet most studies have examined just one or a few species (Woodward *et al*. 2010a). There are three routes by which an overall community size difference between systems at different temperatures or different levels of another abiotic factor could be realized (Fig.1), and to our knowledge the relative contributions of these routes to size shifts in actual communities have never been determined. These routes are conceptually similar to those outlined by Daufresne *et al*. (2009) at the population and community levels. First, *intraspecific size shifts* (Fig.1b) occur when the body sizes of individual species respond similarly to temperature, as in James’ rule, such that the response will be reflected in a shift in the community mean body size. The other two mechanisms are types of compositional shift, as in Bergmann's rule. These can be manifested by s*pecies relative abundance differences* (Fig.1c), where species of different mean size differ in relative abundance in systems at different temperatures, or in the more extreme form of *species turnover* (Fig.1d) due to local extinctions or invasions. Turnover may occur spatially among systems along a temperature gradient (e.g. after O'Gorman *et al*. 2012) or temporally, if warming occurs over time. The second goal of this article will be to assess the relative importance of these three contributors to community size shifts in diatoms.

**Figure 2:**
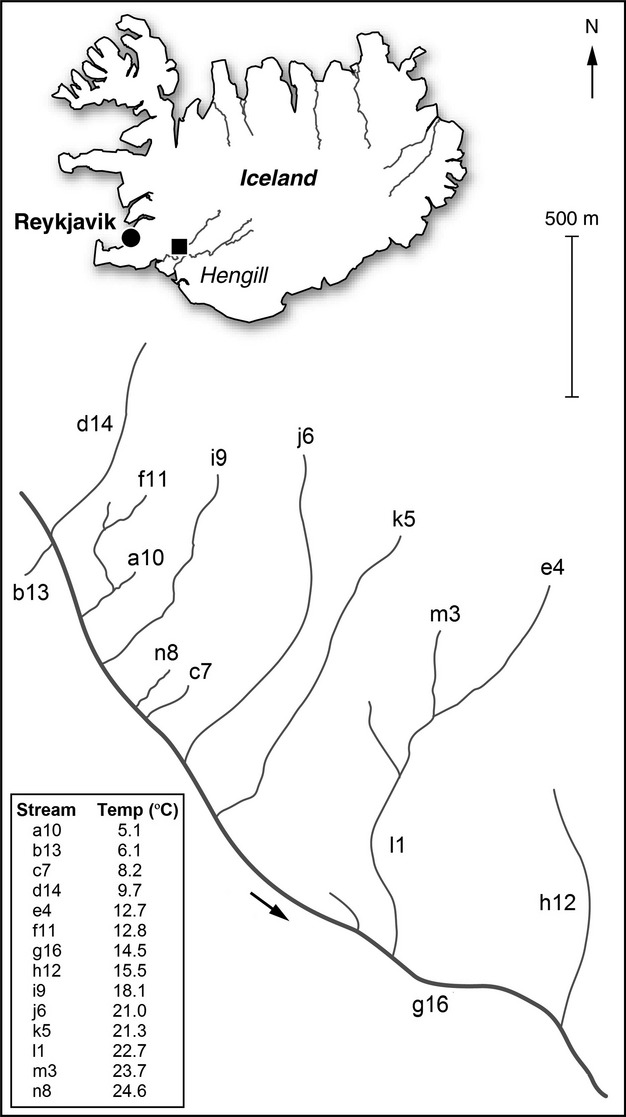
Map of the Hengill system within Iceland. Streams used are labelled using the numbering system outlined in Methods. The table shows mean August 2008 temperatures for the streams. The arrow is water flow direction.

The few studies that have examined phytoplankton or phytobenthos community responses to warming have had one or both of two common limitations that our study avoids: either they have been conducted in controlled mesocosm or microcosm environments (Lewandowska & Sommer 2010; Rüger & Sommer 2012), which have limited realism (Friberg *et al*. 2009), or they have been conducted over large latitudinal (Morán *et al*. 2010) or temporal (Winder *et al*. 2009) scales, introducing dispersal constraints and confounding environmental covariates. To overcome these limitations, we used a system of geothermally heated streams in Iceland that span a 20 °C difference in temperature whilst being no more than 2 km apart (Fig.2). The diatom assemblages of 14 streams within this catchment were sampled *in situ*, providing a rare natural experiment that allows us to capture the effects of warming on real, complex systems, without the confounding effects introduced by biogeography, and with minimal possibility for confounding effects of other environmental variables besides temperature (see Methods, Table S1 and Fig. S1 for more details; Woodward *et al*. 2010b). Previous studies carried out at this site (e.g. Friberg *et al*. 2009; Woodward *et al*. 2010b; O'Gorman *et al*. 2012) and at other geothermal sites (e.g. Lamberti & Resh 1985) have shown that this form of warming is a reasonable proxy for climate change.

Here, we address two specific questions for each of our two research goals: (ia) For each species, how is mean body size per stream related to stream temperature, and how consistently is decreased size observed at warmer temperatures? (ib) How is mean body size of the diatom community in each stream related to stream temperature? (iia) What is the relative importance of intraspecific body-size shifts and species compositional shifts in producing whole-community size shifts (i.e. the relative importance of Bergmann's rule and James’ rule)? (iib) What is the relative importance of species relative abundance shifts and species turnover effects with temperature? This study is the first we know of both to address systematically the nature of size shifts for multiple diatom species in a natural ecosystem, and to examine the relative importance of both intraspecific and species compositional changes for community size shifts.

## Materials and methods

### Study site

The study was done in the geothermal Hengill region of Iceland (64°03′N, 021°18′W, 350–420 metres above sea level). The area contains multiple indirectly heated streams that are tributaries to the same main stem and which all lie within 2 km of one another, such that dispersal constraints are negligible across the whole system (Fig.2; see O'Gorman *et al*. 2012 for a more detailed site description). Stream water temperatures are altered by geothermal warming from deep below the streambed. Temperature is the major determinant of the taxonomic composition of assemblages of both primary and secondary producers across the streams (Woodward *et al*. 2010b; Gudmundsdóttir *et al*. 2011). These features enable us to isolate the effects of temperature on diatom community composition and size structure. Fourteen streams in the system were used for this study (Fig.2), spanning about 5–25 °C. This stream system was the subject of previous work (Woodward *et al*. 2010b; Gudmundsdóttir *et al*. 2011; O'Gorman *et al*. 2012), in which the streams were named numerically. We have changed notation slightly so readers can more easily distinguish between warm and cold streams. Streams we used are labelled with the letters a-n in order from the coldest to the warmest stream, followed by the number used in the original system, so that readers can easily compare with previous work.

### Environmental variables other than temperature

A broad suite of physical and chemical variables was measured in the streams in August 2008, concurrently with the diatom sampling (see Woodward *et al*. 2010b; Demars *et al*. 2011; O'Gorman *et al*. 2012 for detailed methods). These variables included the major macronutrients (e.g. nitrate, ammonium, orthophosphate), ions (e.g. Ca^2+^, K^+^, Na^+^, Cl^−^), trace elements (e.g. Si), pH and conductivity (see Table S1). Besides temperature and a few variables directly linked to it by physico-chemical processes, all water chemistry and environmental variables were broadly similar among the streams and not strongly related to temperature (Friberg *et al*. 2009; Woodward *et al*. 2010b; Demars *et al*. 2011; O'Gorman *et al*. 2012; Table S2). Among those that are driven by physical laws that apply to all systems, dissolved oxygen concentrations declined with temperature, but the % saturation was independent of temperature across the gradient. The other two exceptions are silicon (Si) and K^+^ concentrations, which increased with temperature, reflecting the increased chemical weathering of rocks. These two abiotic variables are unlikely to have ecological significance for the diatom assemblages at Hengill because Si is found in concentrations at least one order of magnitude above what is typically considered limiting in freshwaters (White *et al*. 1999; Dalai *et al*. 2002; Dupré *et al*. 2003) and potassium (K) is thought to be unlikely to limit growth in natural waters (Hynes 1970; Jaworski *et al*. 2003; Talling 2010). Algal production in these streams is (co)limited by N and P (Friberg *et al*. 2009), as is typical of freshwater ecosystems, but concentrations of these macronutrients were not strongly related to temperature and there was very little variation across the catchment: total variation was <0.1% of the range for European streams (Woodward *et al*., 2012 and also Figure S1).

### Diatom sampling

Sampling took place in August 2008. Five stones were selected randomly from each stream. Potential in-stream and between-stream habitat heterogeneity was minimized by sampling from riffles. Stones were representative of the typical size of cobbles on the surface of the armoured layer and were similar sizes in each stream. Algal scrapes were collected from the whole upper (projected) surface of each stone using a toothbrush. The stone area sampled was recorded and samples were analysed separately. Samples were preserved immediately in Lugol's solution, and a fixed volume of the sample solution was subsequently acid-digested and mounted on microscope slides (after O'Gorman *et al*. 2012). Relative abundance of diatom species was determined by counting individuals present in a 100 μm wide transect across the centre of the slide and calculating 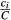 where *C* is the total number of individuals counted per stream, and *c*_*i*_ is the total number of individuals found of species *i*.

### Diatom identification and measurements

Diatom species that accounted for the top 95% of abundance in each stream were measured for size: the remainder species were grouped together and designated as a single group, ‘other’, for later use in resampling schemes. There were several species outside the 95% abundance group but these were always very rare. Within each stream, for every species and for the ‘other’ group, ten individuals per group were selected for size measurement along a transect starting at the centre of the slide, using an Olympus BH2 microscope. Samples for the ‘other’ group were taken as the first ten individuals found on a slide that were outside the top 95% for that stream. For some groups, 10 samples could not be found; in those cases as many were found as possible. Samples from ‘other’ were also identified to species level. Broken or obscured valves were not used. Individuals selected for size measurement were photographed in valve view with a high resolution Canon digital SLR camera. Any individuals that were lying out of focus had multiple images taken and were rendered in Helicon Focus (Helicon Soft Ltd, 2011). Images were taken at a magnification of ×1000, except for a few very large individuals where ×400 was used. Diatoms were identified using the species definitions and nomenclature of Krammer and Lange-Bertalot (1986–1991). Classifications beyond the species level were not used.

Measurements were taken in Image J (Abramoff *et al*. 2004) for valve length and maximum valve width. Diatom size is sometimes estimated using cell volume, using dimensions taken from diatoms in both valve view and girdle view (Hillebrand *et al*. 1999). In this study, projected (cross-sectional) valve area was used because measurements of individual diatoms were required, and because each single diatom was lying in either valve or girdle view. This is a reliable measurement of body size as diatom cell volume is strongly correlated with valve length (Snoeijs *et al*. 2002; Finkel *et al*. 2009b) and valve area incorporates valve length as well as the additional information of valve width, and hence is likely to be even better correlated with volume. Valve area (μm^2^) was calculated using a standard geometric shape for each genus as described in Hillebrand *et al*. (1999), with a few exceptions where the shape of the species in the Hengill region was distinctly different from the described shape for the genus. Table S3 and Figure S2 justify the exceptions, showing that our shape modifications improved accuracy.

### Statistical analysis: intraspecific patterns

To determine the relationship between temperature and body size within individual species, we considered every species that was present in two or more streams. Linear regressions were carried out for each species, of temperature against body size of individuals. A Bonferroni correction for multiple tests was applied: *P*-values were multiplied by 31, the number of tests. Size data were log_10_-transformed for this and all analyses.

During the asexual part of their life cycle, diatom cells progressively reduce in size (Edlund & Stoermer 1997). Since we have used a natural experiment where the sampled individuals could be at any stage of their life cycle, this phenomenon could potentially be contributing to any cell size differences we observe. However, this vegetative cell size reduction is not uniform and in pennate diatoms the width of the valve decreases proportionally far less than the length (Round *et al*. 1990). Thus, we repeated the intraspecific analysis using only valve width as the measure of body size, as a way of determining whether the diatom life cycle could have confounded our results.

### Statistical analysis: whole-community analysis

Average diatom size for each stream was calculated using a simple weighted mean. If *k*_*α*_ is the number of species present in stream *α* (including the ‘other’ group as a single ‘species’ for this count), *n*_*αi*_ is the number of samples taken of species *i* (cells measured, usually 10), *a*_*αi*_ is the relative abundance of species *i* in stream *α*, *s*_*αij*_ is the log_10_ size measured for individual *j* of species *i* and 
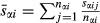
 is the average log_10_ size of species *i* in *α*, then the abundance-weighted mean size for *α* is 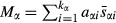. The *a*_*αi*_ are relative abundances, so they sum to 1 across all species (including ‘other’ as a species). Confidence intervals of the abundance-weighted mean were calculated using a resampling method (Crowley 1992); for each stream, we calculated *M*_*α*_ for a resampled data set 10 000 times and from those took 95% confidence intervals, as follows. The resampled data set was obtained by first resampling the original size data for each species *n*_*αi*_ times with replacement (including ‘other’ as a species), and second, recalculating the relative abundance of each species by resampling, with replacement, the original diatom count data *C* times using multinomial trials with probabilities proportional to the *c*_*i*_ for the species (including ‘other’).

### Statistical analysis: partitioning the causes of community size change

We partitioned the causes of community size change in two ways: first, by determining whether intraspecific effects or compositional shifts are the main contributor to whole-community size differences; and second by determining if compositional shifts are mostly due to differences in relative abundances of species or species turnover.

We determined the proportional contribution of intraspecific effects to whole-community size differences by calculating the size difference we would expect between any two streams, *α* and *β*, if there were no differences in community composition, and comparing this to the observed size differences between *α* and *β* in average diatom size (computed as described above). To remove compositional effects, we first considered only species that were present in both *α* and *β*. To remove effects of differences in the relative abundance of species between *α* and *β*, which is a compositional effect, *a*_*αβi*_ was calculated as 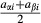, the average relative abundance of species *i* in *α* and *β*. Then, if *k*_*αβ*_ is the number of species in common between *α* and *β*, the size difference we would expect due only to intraspecific effects is

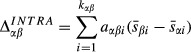


The overall average community size difference between streams *α* and *β* is 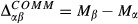 and so the proportional contribution of intraspecific effects is 
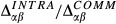
. In the case where there are no compositional differences between streams *α* and *β*, 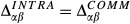. 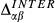 was calculated as 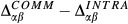.

There are three possible outcomes to this analysis. If the result is negative then the intraspecific size difference was in a different direction to the overall community size difference and so the observed direction of body-size difference between *α* and *β* is solely due to compositional shifts which overcame contrasting shifts in individual species; in other words, 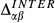 had opposite sign and larger magnitude than 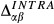. If the result is 1 or above, then the overall community size difference is solely due to intraspecific size differences which overcame competing compositional shifts; in other words, 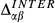 had opposite sign and smaller magnitude than 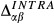. If the result is between 0 and 1 then the overall community size difference is due to a mixture of intraspecific size differences and compositional shifts, and the value indicates the proportional contribution of 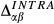. In that case, 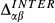 and 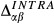 have the same sign.

To provide illustration of the methods using simplified two-species examples, Table1 gives calculations of 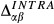 and 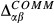 for comparisons between a reference stream (*α*) and other streams (*β*_i_); the three outcomes described above are shown. In all cases, the average community body size decreases from stream α to stream *β*_i_ and 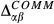 is negative, but the causes of the decrease are different. Examples (a) and (b) show the simplest examples, where only one cause of community size change is contributing. In (a), there is no change in the relative abundance of species between streams, only changes in the log_10_ size, therefore only intraspecific effects are contributing to the community size difference and 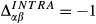 and 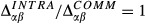. In (b), there is no change in the log_10_ size of species, only in the relative abundances of the species; therefore, only compositional effects are contributing and 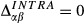 and 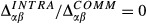. Examples (c) to (f) illustrate a mixture of causes contributing to the difference in average community size between the streams. In (c), there is a decrease in the log_10_ size of each species, and also a shift in the relative abundance towards species 1, the smaller species. Therefore, both intraspecific and compositional effects are occurring in the same direction. 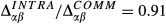, so intraspecific effects are responsible for 91% of the average size difference between streams. In (d), there is the same decrease in log_10_ size as in (c) but the shift in the relative abundance is in the opposite direction towards species 2, the larger species. In this case, intraspecific effects and compositional effects are acting in opposite directions, so 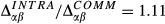: intraspecific effects overcame compositional effects and are the only cause of the overall size decrease. In (e), there is an increase in log_10_ size of species 2, and a shift in relative abundance towards species 1. This is the opposite of example (d): 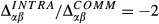, and compositional effects overcame intraspecific effects. Example (f) shows the same outcome from the analysis as (e) with 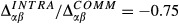 but in this case the compositional effects are due to species 3, a smaller species, replacing species 1. Turnover is the cause of the size decrease between the streams, rather than relative abundance shifts as in (e).

**Table 1:** Examples of calculations for 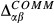 and 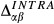 between streams *α* and *β*_j_. Each example illustrates a difference from stream α in either (i) a change in log size for one or both species; (ii) a shift in the relative abundances of the species; (iii) a different species being present or (iv) a combination of these. 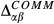 is calculated for a two–species system as 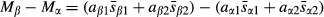, where *a*_*αi*_ and *a*_*βi*_ are the relative abundances of species *i* in streams *α* and *β*_._ 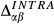 is calculated as 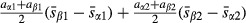. There is only one species in common between *α* and *β*_6_ so 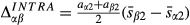

		Species 1		Species 2		Species 3					
	Stream	abundance	log_10_ size ( 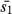 )	abundance	log_10_ size ( 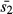 )	abundance	log_10_ size ( 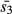 )	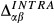	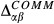	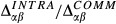	Result
	*α*	7	2	3	3	–	–				
(a)	*β*_1_	7	1	3	2	–	–	−1	−1	1	ALL INTRA
(b)	*β*_2_	8	2	2	3	–	–	0	−0.1	0	ALL COMP
(c)	*β*_3_	8	1	2	2	–	–	–1	−1.1	0.91	91% INTRA
(d)	*β*_4_	6	1	4	2	–	–	–1	−0.9	1.11	ALL INTRA
(e)	*β*_5_	9	2	1	4	–	–	0.2	−0.1	−2	ALL COMP
(f)	*β*_6_	–	–	3	4	7	1	0.3	−0.4	−0.75	ALL COMP

To assess the types of compositional shift that may be contributing to differences in body size we considered the presence and absence of species (all species found in the streams, including those in the ‘other’ group) between streams and their relative abundances in the streams. We calculated the proportion of compositional change from stream *α* to stream *β* that was represented by species turnover, as opposed to species relative abundance shifts, as the sum of the relative abundances of the species that were present in *α* but not detected in *β*. If the main compositional difference between streams is mostly because of species turnover, then these values will be close to 1. If the main compositional differences are due to relative abundance shifts, then the values will be close to 0.

Intraspecific effects are the main contributor to changes in overall community size differences from one stream to another when the index 
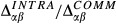
 is greater than 0.5, and species turnover is the main contributor to compositional shifts when the index of the previous paragraph is greater than 0.5. Thus, the total fraction of all possible pairwise comparison values among streams that were greater than 0.5 in each analysis indicates the number of cases in which intraspecific effects or species turnover, respectively, are relatively more important, and characterizes the importance of these effects in the whole system. We calculated confidence intervals for these measures using a resampling method. Data were resampled 1000 times according to the same scheme used for calculating confidence intervals for average community size, and all pairwise comparisons between streams were re-calculated for each surrogate data set created in this way. For each resampling, the total number of comparisons that showed a>50% contribution of 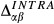, or species turnover, was counted, and 95% confidence intervals for these numbers were taken as quantiles from the resulting distributions. All computations were carried out in the R programming language (version 2.14.1; R Development Core Team 2010).

## Results

Overall, 75 species were detected across the 14 streams of the study, and a total of 20 835 individual diatoms were counted to obtain relative abundance estimates and 2006 diatoms were measured for body size. Complete data are included in Tables S1, S10, S11.

### Intraspecific size changes

There were 31 diatom species present in two or more streams, and these were used in the intraspecific analysis. Temperature appeared to have a significant effect on the body size of some diatoms. The slopes of intraspecific regressions (Table2, Figure S3) revealed that the effect of temperature on log_10_ body size was species-specific, with increasing temperature having a negative effect in 16 cases (seven significant with *P* < 0.05) and a positive effect in 15 cases (five significant with *P* < 0.05). Seven relationships were significant at the 5% level after Bonferroni correction: 3 negative slopes (corrected *P* = 0.0022, *P* = 0.0114 and *P* = 0.0266) and 4 positive slopes (corrected *P* = 0.0026, *P* = 0.0015, *P* = 0.0101 and *P* = 0.0029). If species were excluded which only occurred in two streams, there were 26 species remaining, 15 with negative slope (3 significant with *P* < 0.05) and 11 with positive slope (3 significant with *P* < 0.05). Five relationships were significant at the 5% level after Bonferroni correction, 3 negative and 2 positive. If species were excluded which only occurred in three or fewer streams, 24 species remained, 14 with negative slopes (3 significant) and 10 with positive slopes (3 significant). Again, 5 were Bonferroni significant, 3 negative and 2 positive. Regardless of the details on how significance is judged or including vs. excluding species that occurred in few streams, results were about evenly divided between species with increasing and decreasing slopes: no consistent influence of temperature across species was apparent.

**Table 2:** Summary of slopes and *P*-values from intraspecific body-size vs. temperature analysis for 31 diatom species. num.streams is the number of streams in which each species was found; since most species’ body sizes were measured for 10 individuals per stream, the number of points used in each regression was close to 10 times num.streams. Corrected *P*-values are Bonferroni corrected. Authorities for these and all species names are listed in Table S12

Species	num.streams	Slope	*R*^2^	*P*-value	Bonferroni corrected *P*-value
*Achnanthes lanceolata*	13	−0.0014	0.0039	0.48597845	1
*Amphora inariensis*	6	−0.0084	0.1114	0.01116087^*^	0.34598697
*Amphora pediculus*	6	−0.0046	0.0842	0.02450351^*^	0.75960881
*Caloneis lauta*	2	0.0029	0.0123	0.64100834	1
*Cocconeis pediculus*	4	0.0379	0.0849	0.08944546	1
*Cocconeis placentula*	10	−0.0013	0.0017	0.68446086	1
*Cymbella sinuata*	2	0.0325	0.5866	0.00008266^***^	0.00256246^**^
*Diatoma mesodon*	7	−0.0091	0.0879	0.01267111^*^	0.39280441
*Epithemia sorex*	3	−0.0097	0.0598	0.20974975	1
*Epithemia turgida*	8	−0.0003	0	0.96022789	1
*Fragilaria arcus*	2	0.0259	0.6105	0.00004755^***^	0.00147405^**^
*Fragilaria capucina*	14	0.0014	0.0061	0.35911286	1
*Fragilaria construens*	2	0.0338	0.097	0.2083153	1
*Fragilaria pinnata*	8	0.002	0.0076	0.48555925	1
*Gomphonema clavatum*	4	0.0125	0.345	0.00032597^***^	0.01010507^*^
*Gomphonema clevei*	4	0.0066	0.0986	0.07517861	1
*Gomphonema parvulum*	6	−0.01	0.0355	0.25732075	1
*Gomphonema type D*	8	0.0015	0.0065	0.50645964	1
*Melosira varians*	8	0.0343	0.1896	0.00009458^***^	0.00293198^**^
*Meridion circulare*	10	−0.0117	0.1618	0.00007049^***^	0.00218519^**^
*Navicula atomus*	4	−0.0007	0.0017	0.79776186	1
*Navicula minima*	13	0.0005	0.0011	0.71545164	1
*Navicula placentula*	6	−0.0091	0.2076	0.00036827^***^	0.01141637^*^
*Nitzschia dissipata*	4	0.0122	0.0242	0.37999347	1
*Nitzschia fonticola*	2	−0.0458	0.0645	0.32545121	1
*Nitzschia inconspicua*	3	0.0147	0.1694	0.08971087	1
*Nitzschia palea*	8	0.0096	0.0857	0.00885657^**^	0.27455367
*Nitzschia paleacea*	9	−0.0061	0.0336	0.09739664	1
*Rhoicosphenia abbreviata*	5	−0.0298	0.2253	0.00085866^***^	0.02661846^*^
*Rhopalodia gibba*	5	−0.0154	0.1626	0.00406858^**^	0.12612598
*Synedra ulna*	10	−0.0012	0.0016	0.72143697	1

Stars represent levels of significance: *P* < 0.05^*^, *P* < 0.01^**^, *P* < 0.001^***^.

Because results showed effects in opposite directions, to see whether there was a significant average effect of temperature on body size among species, we calculated a combined *P*-value for the 31 regressions using a weighted Stouffer's method (Stouffer *et al*. 1949; Whitlock 2005). Under a null hypothesis of diatom species not reducing in body size with increased temperature, the combined *P*-value was not significant (*P* = 0.186), so there was no average tendency for species to decrease in size with increasing temperature. If species that occurred in only two streams were excluded from the analysis, the combined *P*-value was still not significant (*P* = 0.113), nor was it significant if those that occurred in three or fewer streams were excluded (*P* = 0.112). Two-tailed analogues of the above tests, which tested the null hypothesis that diatom species’ sizes do not depend systematically on temperature, on average, had double the *P*-values reported above (Whitlock 2005) and hence were also not significant. Thus, there was no overall tendency for species to decrease or increase in size with increasing temperature.

The repeated intraspecific analysis using only valve width as the measure of body size gave similar results (Table S4), so the lack of clear and consistent body-size trends we observed using valve area could not be ascribed to an artefact of the diatom reproductive cycle.

### Whole-community size changes

**Figure 3:**
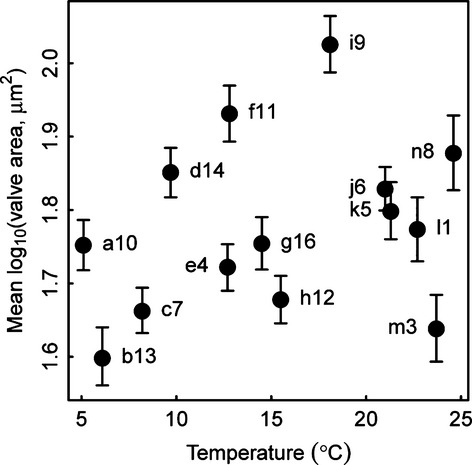
Mean body sizes of diatoms in the fourteen sampled streams, with 95% confidence intervals of the means, plotted against temperature.

Figure3 shows that there is no clear relationship between temperature and body size at the community level. Both linear and quadratic regressions of mean log_10_ valve area against temperature, with weighting by inverse variances of mean log_10_ valve area estimates applied, were not significant (*P* = 0.2628 and *P* = 0.3785 respectively). In Fig.3, streams h12 and m3 appear to be possible outliers, but repeating the linear and quadratic regression analyses without those streams also gave insignificant results (*P* = 0.0981 and *P* = 0.1742 respectively). Thus, there was no strong effect of temperature on community body size for diatoms in these streams, contradicting Bergmann's rule. If body-size shifts in diatoms were a strong and general pattern, then across the large temperature gradient we have used (ca. 20 °C) we would expect to see significant relationships in these results for community-level and species-level patterns. The fact that we do not contradicts the supposed universality of the temperature–size relationships.

### Intraspecific effects vs. compositional shifts

**Figure 4:**
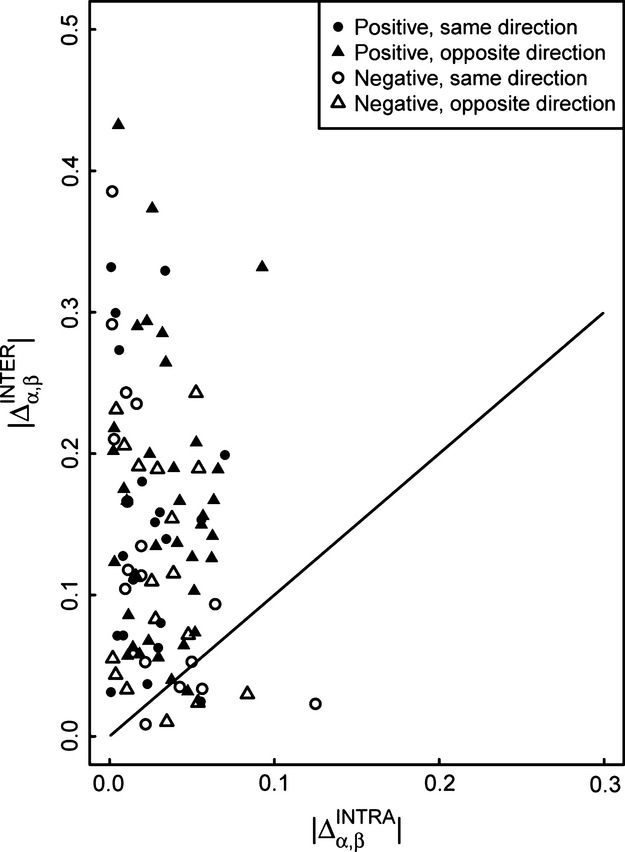
Relative importance of intraspecific and interspecific shifts in producing overall community size shifts in pairwise comparisons among streams, represented using 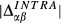 vs. 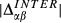 for 91 pairwise comparisons. Recall that 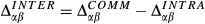. A 1:1 line divides the plot; points above the line represent pairwise comparisons where interspecific effects were the greater contributor to community size differences, and points below the line represent pairwise comparisons where intraspecific effects were the greater contributor. Triangles represent pairwise comparisons where intraspecific and interspecific effects were acting in opposite directions, and circles where they were acting in the same direction. Solid points represent pairwise comparisons where 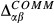 was positive, so body size increased with temperature between the two streams, and open points where 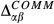 was negative.

Compositional shifts were the main causes of the overall differences in average community body size. Of 91 pairwise comparisons between average community body size in the fourteen streams (Table S5), 51 showed that the observed difference in average community body size was solely due to compositional shifts (negative values of 
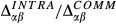
), and only four showed that the difference was solely due to intraspecific size change (values equal to 1 or above). Thirty-six were due to a mixture of the two mechanisms (values between 0 and 1), but of these 36, the average contribution of intraspecific size change to overall community change in body size was 21.8%, with a range from 0.28% to 84.4%. Only five of these 36 comparisons showed that intraspecific size differences were the main mechanism (over 50% contribution), and therefore only nine of 91 comparisons, total, indicated a greater role for intraspecific effects than compositional shifts; 95% confidence intervals for this count based on resampling were 7 to 16, the entire range of which was much less than half of the 91 pairwise comparisons made. In 55 of the comparisons, intraspecific and interspecific effects were acting in opposite directions, and in 36 they were acting in the same direction. Fig.4 shows that intraspecific effects were the dominant contributor to community body size in only nine of the 91 comparisons, and this mostly occurred when the direction of the size shift between the streams was negative.

### Relative abundance shifts vs. species turnover

Species relative abundance differences were the main cause of compositional shifts, rather than species turnover. Pairwise comparisons between streams (Table S6) showed species turnover contributed 0.5–71.7% of the difference in species composition, and the average contribution of species turnover was 14.8%. Out of 182 comparisons, only 34 showed species turnover contributing>25%, and only 6 showed species turnover contributing>50% (95% confidence intervals based on resampling were 6 to 11), and the comparisons with the highest level of species turnover were between streams with larger temperature differences. Because relative abundance differences were the main contributor to compositional shifts, and compositional shifts were the main cause of average community size differences, relative abundance differences were the main contributor to overall community size differences.

### Other abiotic variables

For comparison, we repeated the same analyses done for temperature for many of the other abiotic variables listed in Table S1, although the ranges of variation in those variables were small among our streams (Tables S7, S8 and S9, Figure S4). The comparison with temperature patterns provided additional evidence that temperature does not drive strong, consistent size changes in diatom size. For instance, intraspecific regressions of cell size against pH (Tables S7 and S8) were Bonferroni significant for two positive slopes and two negative slopes; recall that only four positive slopes and three negative slopes were Bonferroni significant using temperature. For silicate, there were five Bonferroni significantly positive slopes and a single negative one. Results were similar for other abiotic variables: temperature was not exceptional either in having an unusually large number of species showing a significant size response nor in the consistency of response directions (positive or negative). Plots of average community diatom size vs. abiotic variables sometimes showed stronger patterns for other variables than they did for temperature (e.g. pH; Table S9 and Figure S4, compared to Fig.3). These results are remarkable because temperature variation across the streams was large and variation in the other abiotic variables was small; if temperature was driving consistent and substantial size variation in diatoms, one would expect observed effects under such circumstances to be stronger for temperature than for the other variables.

We also tested if temperature explained significantly more variation in community mean log_10_ sizes across streams when combined with other abiotic variables than was explained by those other variables alone, finding that it did not. When models containing predictors temperature and pH, temperature and silicate, and temperature and K, respectively, were compared using *F*-tests to models with predictors pH, Si and K, respectively, results were nonsignificant in any case (*P* = 0.347, 0.670, 0.737 respectively). Even though temperature variation across our streams was large and variation in other abiotic variables was small, temperature was less important in driving size variation in diatoms than some of the other variables.

Comparisons between intraspecific effects and compositional shifts and between relative abundance shifts and species turnover were substantially the same regardless of the abiotic variable used. The analogue of Fig.4 in which another abiotic variable is used in place of temperature looks the same as Fig.4 itself, but with the shading of some points changing. Changes just mean a pair of streams, *α* and *β*, in which temperature increased from *α* to *β* happened to show a decrease from *α* to *β* for the abiotic variable used in place of temperature – this change in no way affects the conclusion that compositional shifts were more important than intraspecific shifts. Computations of fractions of relative abundance shifts contributed by turnover also did not depend on the choice of abiotic variable.

## Discussion

In a comprehensive study of diatoms in a natural community, we provided evidence that commonly reported temperature–size relationships do not hold universally for diatoms. At both the intraspecific and community levels, our results do not support the hypothesis of smaller body size under warmer conditions, undermining the generality of Bergmann's and James’ rules and claims that reduced body size is a universal ecological response to warming in aquatic systems (Daufresne *et al*. 2009). Diatoms are a very important exception to these general tendencies because they are abundant, widespread and play a global role in carbon cycling and primary production. Our results also showed that community-level body-size shifts across gradients of temperature or other abiotic variables are determined largely by interspecific effects, with the most important of these being shifts in the relative abundances of species. Intraspecific size changes, which are commonly studied in single-species laboratory experiments, played a much smaller role in the field, highlighting the need for whole-community *in situ* studies for understanding the effects of climate change in natural multispecies systems (Woodward *et al*. 2010a).

At the community level, we found that average diatom cell size does not decrease with temperature. This was unexpected given previous findings in which a shift towards smaller single-celled primary producers with increased warming was observed (Li *et al*. 2009; Winder *et al*. 2009; Morán *et al*. 2010; Lewandowska & Sommer 2010; Yvon-Durocher *et al*. 2011; Peter & Sommer 2012). Our data instead provide evidence supporting some more recent studies that suggest that shrinking body size is not a universal response to increasing temperature (Gardner *et al*. 2011; Rüger & Sommer 2012).

Across the literature there are inconsistent results from studies of individual or few diatom species (intraspecific) responses to temperature, with some studies supporting conformity of diatoms to James’ rule and others claiming they are an exception. Most studies, however, have only considered a small number of diatom or phytoplankton species. Our results go beyond earlier work because of the number of species studied, and therefore provide convincing evidence that diatoms cannot generally or usually be seen to conform to James’ rule. Some older studies show that diatom or phytoplankton cell volume can increase with increasing temperature (Durbin 1977; Thompson *et al*. 1992), but these measured just one and eight species, respectively. More recent studies have shown diatom cell volume decreasing 4% for every °C increase in temperature (Montagnes & Franklin 2001) in a study of ten phytoplankton species (eight diatoms), with similar findings reported by Peter & Sommer (2012) for eleven species of phytoplankton (six diatoms). Both Byllaardt & Cyr (2011) and Rüger & Sommer (2012) concluded that individual diatom species can show an exception to James’ rule, although they measured only three freshwater and seven marine species, respectively. In contrast, our study measured effects of temperature on body size for most of the abundant species present in the diatom community, a total of 31 diatom species. In addition, our study was comprehensive in examining all diatoms that comprised the overwhelming majority of the abundance of a set of natural communities.

It is not well understood why most ectotherms might follow the established temperature–size rules, and several mechanisms have been proposed (Atkinson & Sibly 1997; Partridge & Coyne 1997). Exceptions to general trends, such as those described here, may help illuminate mechanisms. Several mechanisms that may explain shrinking body size are discussed in Sheridan & Bickford (2011), but one of the main explanations put forward invokes changes in the metabolic rate of ectotherms: metabolic rate increases with temperature (Brown *et al*. 2004), so at warmer temperatures, and if resource levels stay the same, growth will be limited and organisms will become smaller to compensate for the increased metabolic demand. However, under these conditions, it seems at least as likely that species will decrease in population size instead of body size, due to increased competition for resources; only if smaller individuals are better competitors for the limited resources, and only if this advantage is accentuated at higher temperatures, would we expect the competitive balance to tip in favour of smaller organisms with warming. It is not clear, *a priori*, whether this is the case in our system, but experimental manipulations could be used to explore this possible explanation of size changes (or lack thereof, in the system of this study). A recent modelling study of Reuman *et al*. (2013) explored the mechanics of a change in relative competitive abilities of differently sized phytoplankton species with warming, parameterizing a nutrient competition and growth model in terms of phytoplankton cell size and ambient temperature. Results suggested that accentuated competitive ability of small phytoplankton cells for nutrient uptake and growth in warmer environments might explain decreased observed plankton sizes in some warmer environments. The model assumed one of several parameterizations for how cell mortality depends on body size and temperature. Whether those assumptions hold in the stream system of this study is not known, but investigating the relationship between that model and conditions in the field may illuminate the explanatory power of the model. Such an investigation may also illuminate the importance of top-down control for helping determine diatom size patterns with temperature in this and other stream systems. The dominant grazers in the system are the snails *Radix peregra* (syn. balthica), which do indeed increase in abundance across the temperature gradient (Woodward *et al*. 2010b; O'Gorman *et al*. 2012). These are very efficient grazers that feed by “bulldozing” entire biofilms in a non-size-selective way; such grazing may favour faster growing species, which tend to be smaller. The model of Reuman *et al*. (2013) also suggests the rate at which non-size-specific grazing mortality increases with temperature may be implicated in whether competition for nutrients among size classes favours larger or smaller cells, or neither, under warming. Thus, further exploration of our system in relation to the model may help illuminate temperature–size patterns in phytoplankton and phytobenthos and variation in these patterns.

Other proposed mechanisms of shrinking under warming relate to alterations in the physical environment caused by temperature change. Winder & Sommer (2012) suggested that phytoplankton will shift towards smaller species because, as water temperature increases and weakens mixing in the water column, small species that sink at a slower rate will be more dominant. This is the explanation given for results in Winder *et al*. (2009), where there is a shift towards smaller species of diatoms over time in lakes experiencing warming. Some previous studies showing a shift towards smaller diatoms examined ocean ecosystems (Li *et al*. 2009; Morán *et al*. 2010) or deep lakes (Winder *et al*. 2009) where stratification may be the most important factor controlling size distributions (Winder & Sommer 2012). Our study sampled benthic (not planktonic) diatoms from shallow streams, where factors such as grazing and competition for resources may be more important, and sinking rates and stratification are irrelevant (Moss *et al*. 2003; Finkel *et al*. 2009a). Hence, the fact that smaller cells were not observed in our warmer streams is consistent with the ideas of Winder & Sommer (2012). Another proposed mechanism for shrinking with warming is that changes in water temperature alter levels of dissolved oxygen, leading to acidification and altered iron levels which may affect phytoplankton cell sizes (Shi *et al*. 2010; Forster *et al*. 2012). Since these chemical changes do not occur in our system, the fact that size shifts are not observed is consistent with the ideas of Shi *et al*. (2010) and Forster *et al*. (2012) .

Most phytoplankton or phytobenthos studies focus on the response of isolated individual species to temperature in the laboratory; however, experimental studies focusing on one or a few species do not provide evidence that global warming favours small diatom species, since the response to temperature seen in laboratory studies is invariably due to intraspecific shifts (Atkinson *et al*. 2003), and these effects were of secondary importance in our natural communities to community-compositional shifts. Therefore, measuring changes in body size of diatom species in natural communities provides a more realistic picture, revealing greater complexity in the community setting. More recently, other studies have started to test the proposed universal rule of Daufresne *et al*. (2009) that there is a shift towards smaller species with increased warming in aquatic communities, with a few considering body-size change of phytoplankton or phytobenthos at a community level (Winder *et al*. 2009; Lewandowska & Sommer 2010; Peter & Sommer 2012; Rüger & Sommer 2012). Future studies should build on the species- and community-level responses of body size to warming demonstrated here, either through natural gradients or experimental manipulations of temperature in real, complex ecosystems, to help disentangle the most important factors determining differences in community structure under warming. Only by understanding the mechanisms underpinning the exceptions to general rules in ecology can we hope to promote true predictability of future climate warming responses.

## Supporting Information

Figure S1 Plots showing a comparison of water chemistry in the Hengill streams to other sites. **Figure S2.** Photographs illustrating shape approximations of a diatom species with an unusual valve outline. **Figure S3**. Linear regression plots of temperature against log_10_ valve area for 31 diatom species that were present in at least 2 of the sampled streams. **Figure S4**. Community-level plots showing mean log_10_ valve area for 8 different abiotic variables in the fourteen streams, analogous to Fig. 3 in the main text. **Table S1**. Water chemistry data for the fourteen streams. **Table S2**. Correlation analysis results of temperature against abiotic variables in the fourteen streams. **Table S3**. Valve areas of thirteen diatom species comparing old and new shape approximations. **Table S4**. Slopes and *P*-values from linear regressions of temperature against log_10_ valve width for 31 species of diatom found in at least 2 of the sampled streams. **Table S5**. Matrix showing the proportion of the total community size change between streams that is due to intraspecific effects. **Table S6**. Matrix showing the proportional contribution of species turnover to overall species compositional differences between streams. **Table S7.** Slopes and *P*-values from linear regressions of 8 different abiotic variables against log_10_ valve area for 31 species of diatom found in at least 2 of the sampled streams. **Table S8**. Summary of significant results in Table S7. **Table S9**. Results from linear and quadratic regressions of temperature and 8 other abiotic variables against mean log_10_ valve area in the fourteen streams. **Table S12**. List of all sampled species names with their respective authorities.

**Table S10**. Size measurements (valve width and length) of diatom individuals sampled from the fourteen streams.

**Table S11**. Count data for all diatom species found in each of the fourteen streams.
