# Supplementary material for: Diatoms can be an important exception to temperature–size rules at species and community levels of organization
Source: Glob Chang Biol. 2013 Aug 18;19(11):3540–52. doi: 10.1111/gcb.12285 (PMC4272575; doi:10.1111/gcb.12285)
Supplement: Figure S1 — Plots showing a comparison of water chemistry in the Hengill streams to other sites. Figure S2. Photographs illustrating shape approximations of a diatom species with an unusual valve outline. Figure S3. Linear regression plots of temperature against log10 valve area for 31 diatom species that were present in at least 2 of the sampled streams. Figure S4. Community-level plots showing mean log10 valve area for 8 different abiotic variables in the fourteen streams, analogous to Fig. 3 in the main text. Table S1. Water chemistry data for the fourteen streams. Table S2. Correlation analysis results of temperature against abiotic variables in the fourteen streams. Table S3. Valve areas of thirteen diatom species comparing old and new shape approximations. Table S4. Slopes and P-values from linear regressions of temperature against log10 valve width for 31 species of diatom found in at least 2 of the sampled streams. Table S5. Matrix showing the proportion of the total community size change between streams that is due to intraspecific effects. Table S6. Matrix showing the proportional contribution of species turnover to overall species compositional differences between streams. Table S7. Slopes and P-values from linear regressions of 8 different abiotic variables against log10 valve area for 31 species of diatom found in at least 2 of the sampled streams. Table S8. Summary of significant results in Table S7. Table S9. Results from linear and quadratic regressions of temperature and 8 other abiotic variables against mean log10 valve area in the fourteen streams. Table S12. List of all sampled species names with their respective authorities. [file gcb0019-3540-sd1.pdf]

Figure S1: Comparison of the range of abiotic variables at Hengill, Iceland (red lines) to observed ranges in 32 other European countries (white bars). Values for 8 Nordic and Baltic states are also shown (grey bars) for a more direct comparison with northern latitude ecosystems. Data on water chemistry from 11,251 running waters from across Europe (sample sizes for each variable are given on each panel) were collated from the European Environment Agency (EEA) database (extracted from databases held at http://www.eea.europa.eu/data−and−maps on 1st April 2013). The figure highlights how Hengill falls well within the range of abiotic variables found across Europe, with only nitrate occurring at the low end of this continental−level gradient.

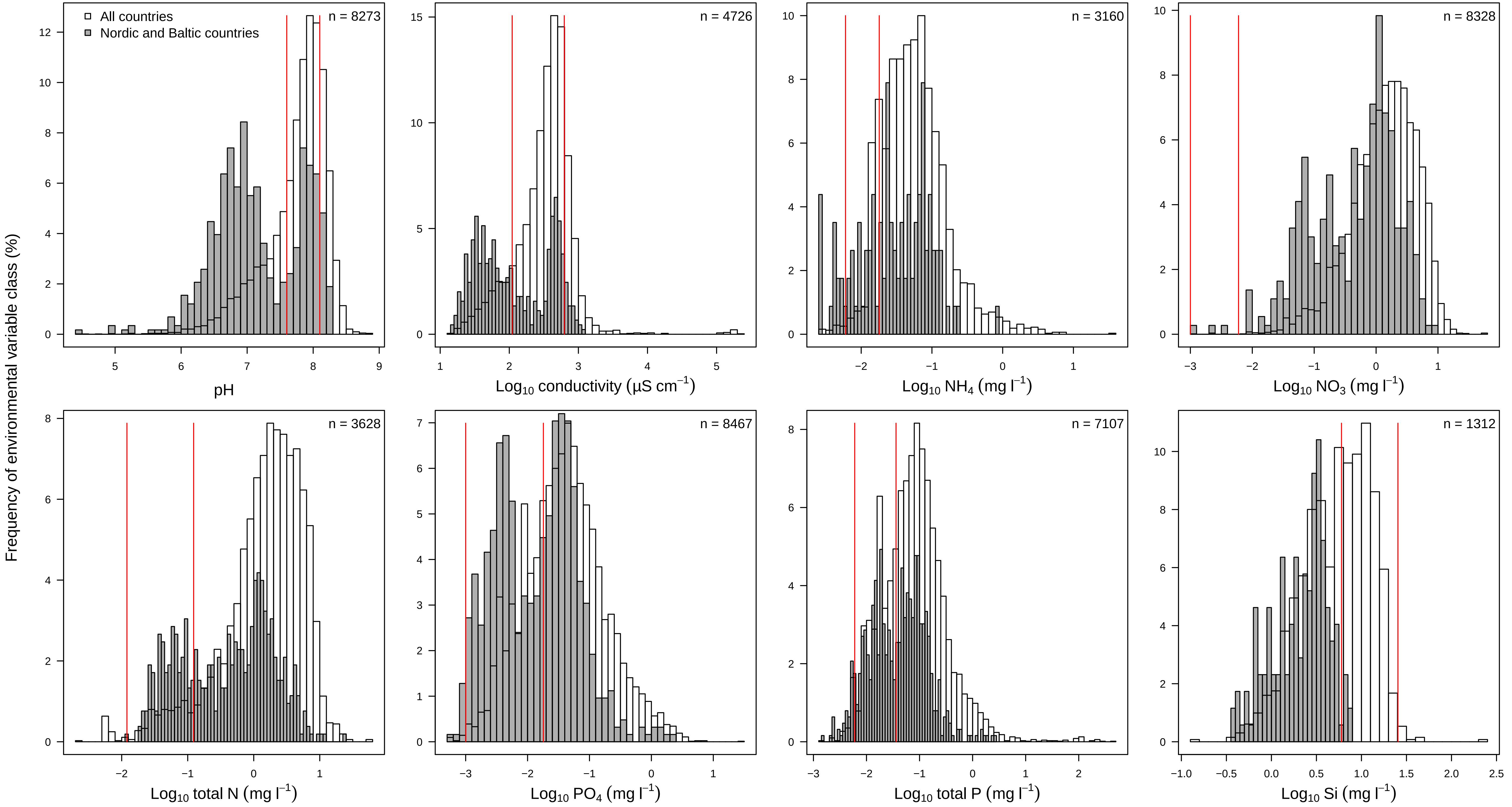

Figure S2: Photographs illustrating shape approximations of a diatom species (*Rhoicosphenia abbreviata*) that showed an unusual valve outline in the Icelandic streams: a) photograph of one individual from stream n8; b) the same photograph with the suggested shape approximation in Hillebrand *et al* (1999) and c) the photograph with the shape approximation used in this study. For every diatom species that showed a shape deviation from the generic shape approximation described in Hillebrand et al. (1999) a more appropriate shape approximation was decided for that species using standard shapes that could easily be calculated using width and length measurements, such as the ellipse shown in (c). To be certain that the new shape approximations were more accurate, we compared the new and old shape approximations with the actual area of the diatom valve for a sample of individuals (one individual per species per stream; Table S3). Actual valve area was calculated in Image J using the outline of the valve; valve area using the shape approximations was calculated using valve width and length measurements.

(a) (b) (c)

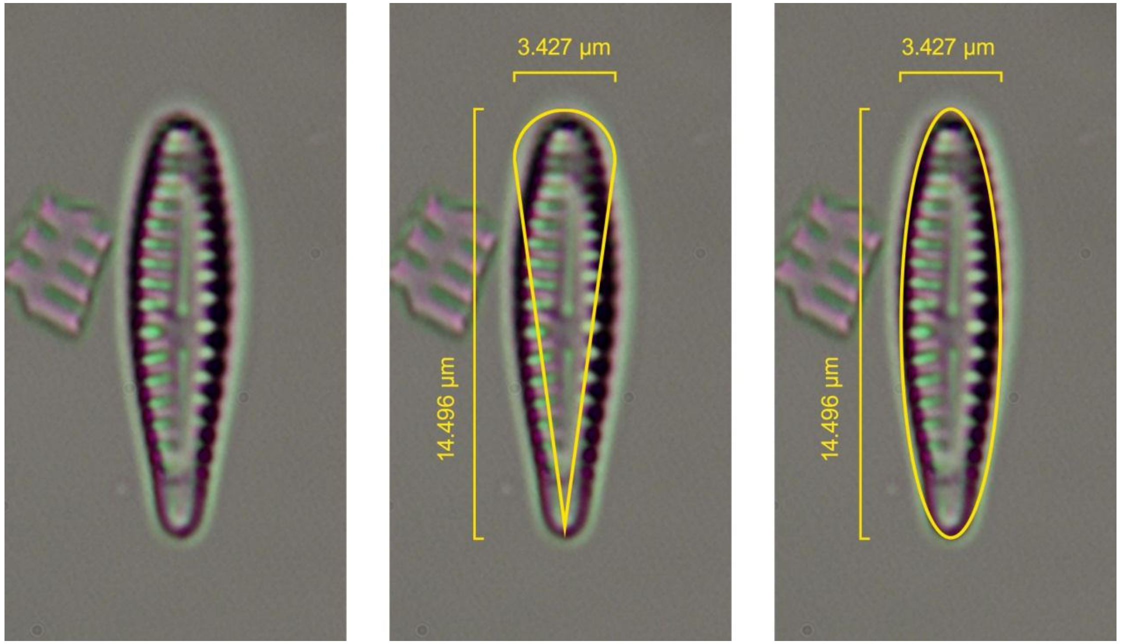

Figure S3: Linear regression plots of temperature against log10 valve area for 31 diatom species that were present in at least 2 of the sampled streams.

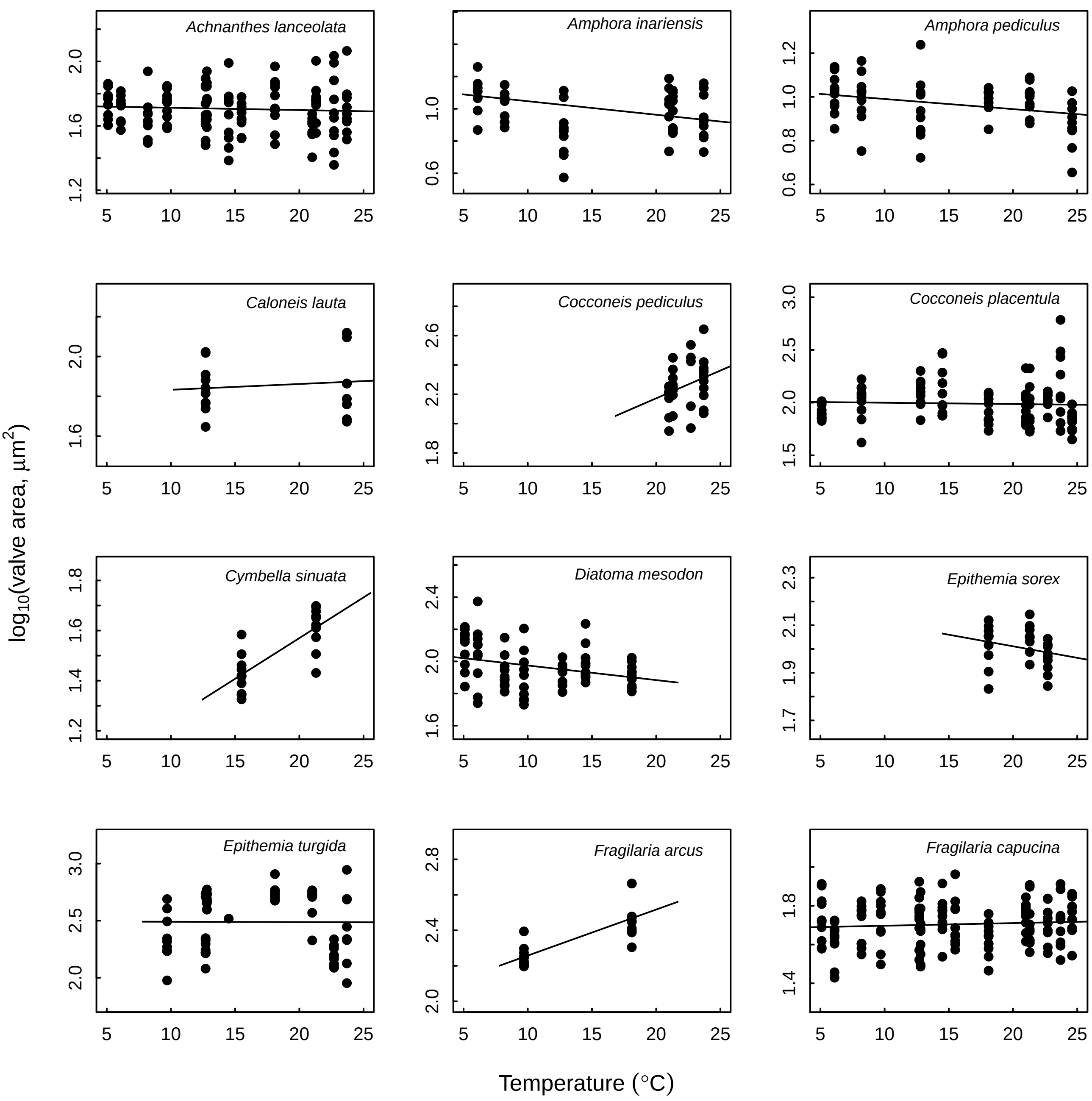

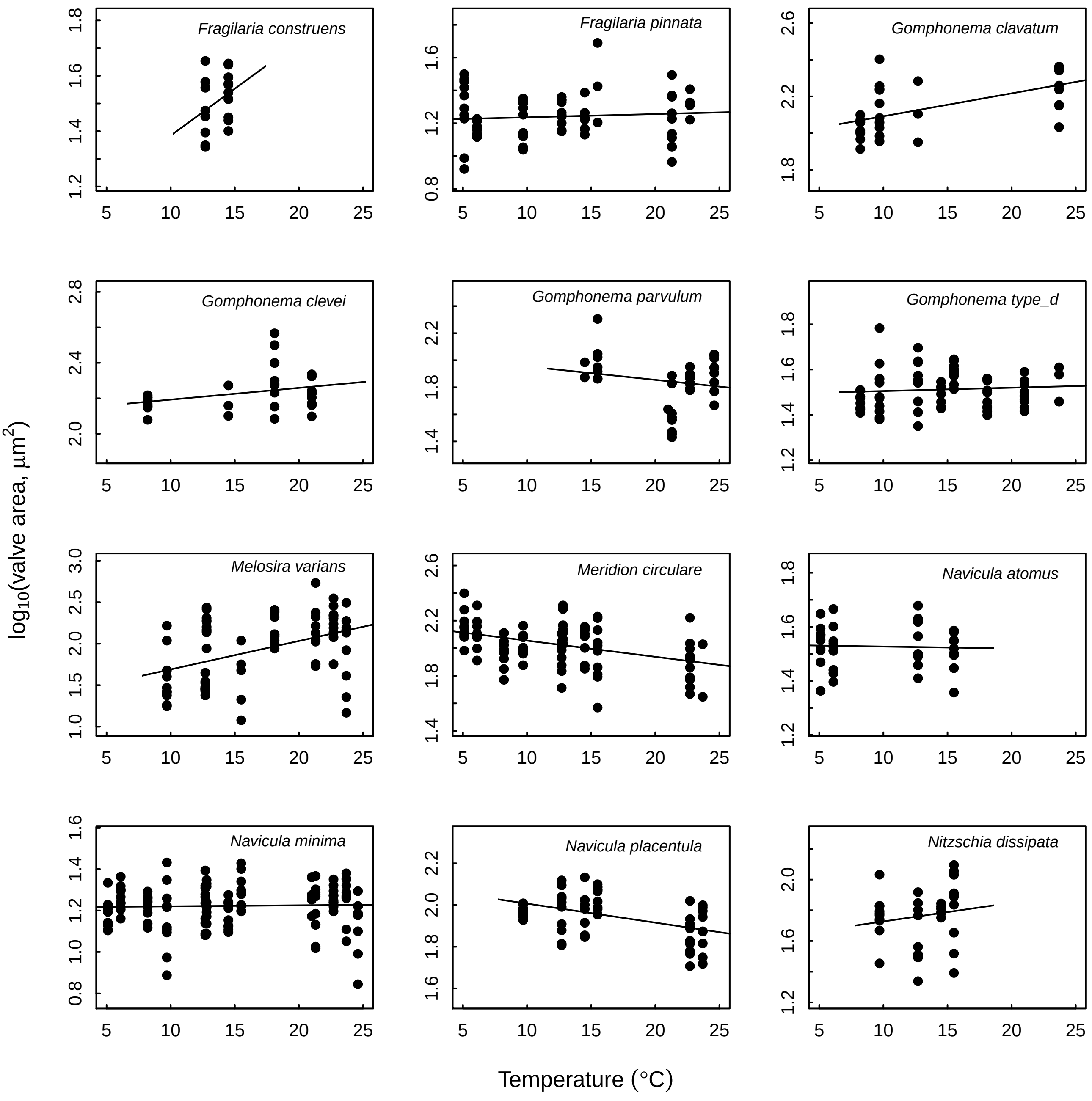
Fragilaria construens
Fragilaria pinnata
Gomphonema clavatum
Gomphonema clevei
Gomphonema parvulum
Gomphonema type_d
Melosira varians
Meridion circulare
Navicula atomus
Navicula minima
Navicula placentula
Nitzschia dissipata
log10(valve area, μm2)
Temperature (°C)

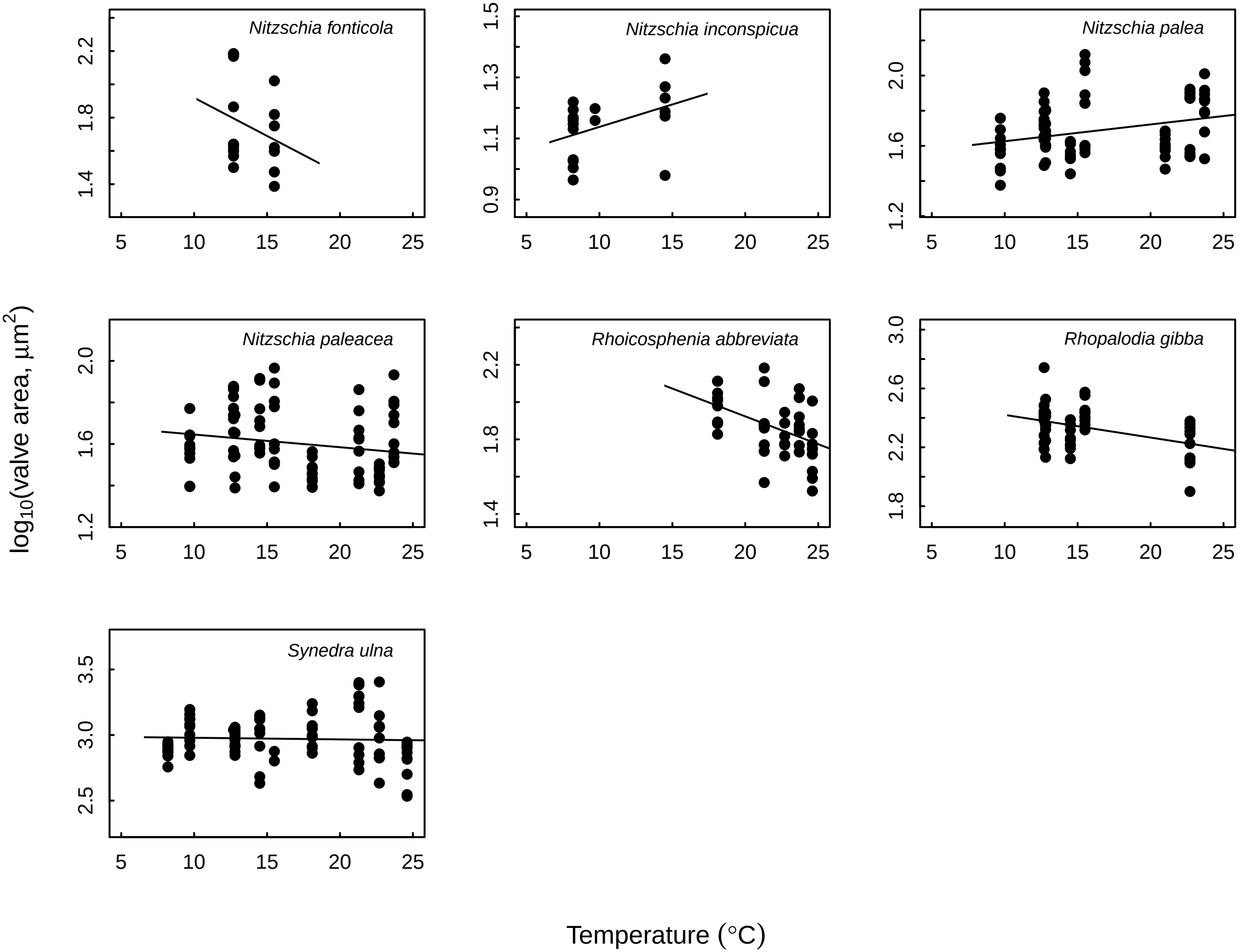
Nitzschia fonticola
Nitzschia inconspicua
Nitzschia palea
Nitzschia paleacea
Rhoicosphenia abbreviata
Rhopalodia gibba
Synedra ulna
log10(valve area, μm2)
Temperature (°C)

Figure S4: Community-level plots showing mean log10 valve area for 8 different abiotic variables in the fourteen streams, analogous to Fig. 3 in the main text.

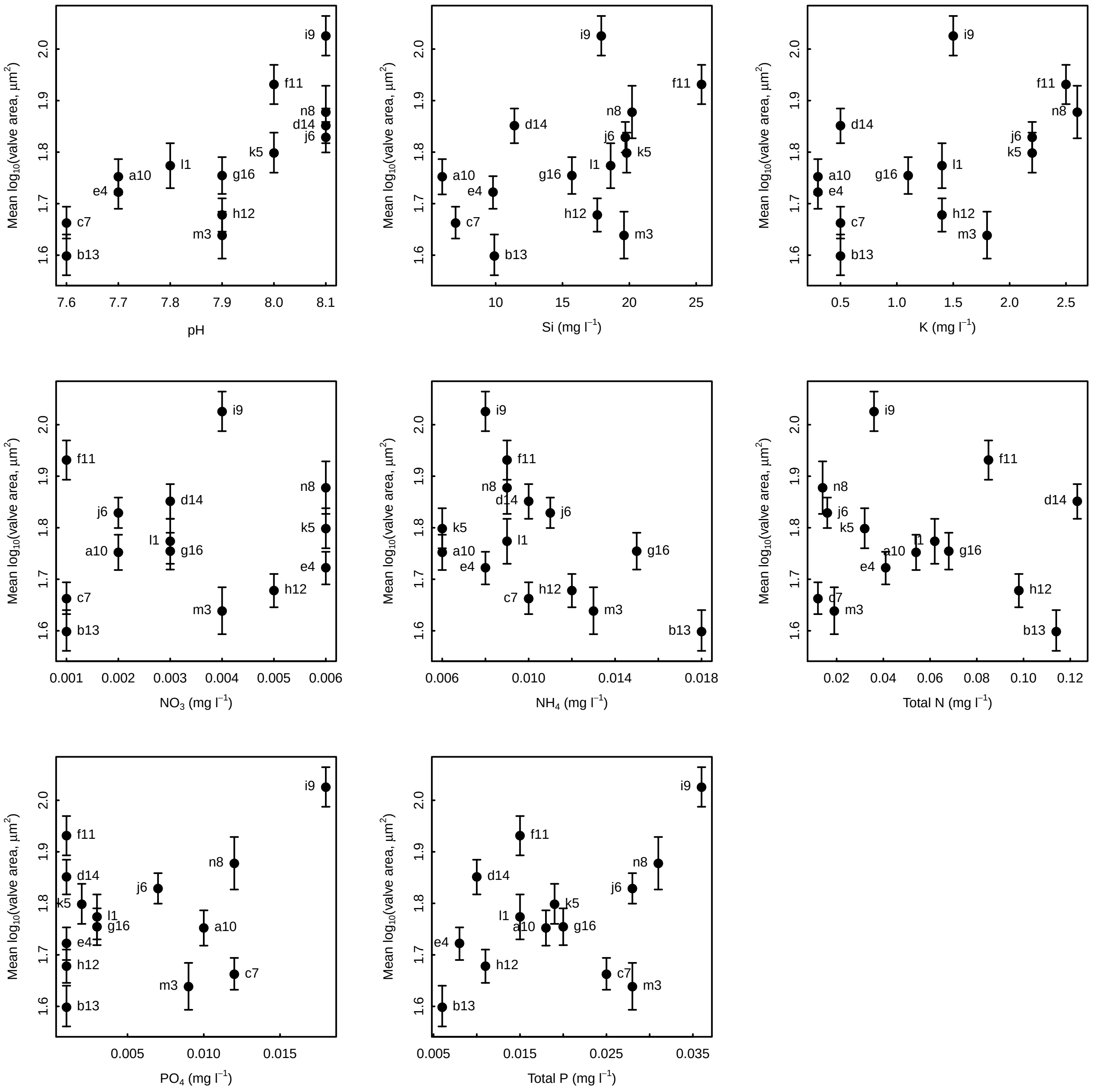

Table S1: Table showing the values for abiotic variables measured in each stream in August 2008, ordered by mean stream temperature. Temp = temperature in °C; pH is unitless; Cond = conductivity in µS cm^{-1}; all other environmental parameters are given in mg l^{-1}. DOC = dissolved organic carbon; DO = dissolved oxygen; TN = total nitrogen; TP = total phosphorous.

Stream	Temp	pH	Cond	DOC	DO	NH_4	NO_3	TN	PO_4	TP	Ca^{2+}	K^+	Mg^{2+}	Na^{2+}	Si	Cl^-	SO_2^{4-}
a10	5.1	7.7	129	0.314	11.7	0.006	0.002	0.054	0.010	0.018	20.1	0.3	4.7	11.0	6.0	6.6	3.8
b13	6.1	7.6	201	0.294	11.1	0.018	0.001	0.114	0.001	0.006	28.4	0.5	8.9	15.1	9.9	7.2	5.9
c7	8.2	7.6	110	0.208	11.4	0.010	0.001	0.012	0.012	0.025	14.1	0.5	4.1	10.6	7.0	7.7	1.5
d14	9.7	8.1	254	0.403	10.7	0.010	0.003	0.123	0.001	0.010	35.9	0.5	8.5	17.5	11.4	6.4	5.8
e4	12.7	7.7	153	0.465	10.7	0.008	0.006	0.041	0.001	0.008	20.3	0.3	5.1	7.4	9.8	7.6	2.9
f11	12.8	8.0	624	0.581	9.9	0.009	0.001	0.085	0.001	0.015	32.4	2.5	29.5	123.8	25.4	5.7	0.2
g16	14.5	7.9	249	0.318	11.1	0.015	0.003	0.068	0.003	0.020	30.5	1.1	8.8	22.0	15.7	6.7	5.8
h12	15.5	7.9	223	0.618	8.1	0.012	0.005	0.098	0.001	0.011	25.1	1.4	7.7	19.6	17.6	7.0	2.6
i9	18.1	8.1	262	0.263	8.5	0.008	0.004	0.036	0.018	0.036	26.9	1.5	6.6	34.6	17.9	6.6	2.7
j6	21.0	8.1	283	0.317	8.6	0.011	0.002	0.016	0.007	0.028	29.8	2.2	6.4	37.0	19.7	6.5	4.6
k5	21.3	8.0	282	0.427	8.0	0.006	0.006	0.032	0.002	0.019	31.1	2.2	6.7	36.8	19.8	6.3	4.8
l1	22.7	7.8	294	0.767	8.2	0.009	0.003	0.062	0.003	0.015	30.8	1.4	7.6	29.0	18.6	6.5	4.9
m3	23.7	7.9	275	0.226	7.1	0.013	0.004	0.019	0.009	0.028	29.5	1.8	6.5	29.9	19.6	6.4	6.4
n8	24.6	8.1	300	0.330	6.8	0.009	0.006	0.014	0.012	0.031	28.2	2.6	5.5	37.5	20.2	6.3	3.5

Table S2: Summary of correlation analysis results for mean stream temperature tested against 16 other abiotic variables measured in the fourteen streams. Cond = conductivity; DOC = dissolved organic carbon; DO = dissolved oxygen. Correlation coefficients (r) are Pearson's correlation coefficients. Corrected p-values are Bonferroni corrected and stars represent levels of significance: p<0.05 (*), p<0.01 (**), p<0.001 (***). (***). P-values were obtained making the standard assumptions of Pearson correlation, but results were similar when p-values were instead calculated by comparing correlation results from data to results from 10000 appropriately randomized datasets.

Abiotic variable	p-value	r	Corrected p-value
pH	0.0255*	0.5927	0.4079
Cond	0.2643	0.3203	1
DOC	0.5218	0.1871	1
DO	2.76e-06***	-0.9219	0.00004***
NH_4	0.5973	-0.1548	1
NO_3	0.0346*	0.5668	0.5530
Total N	0.0569	-0.5196	0.9099
PO_4	0.5096	0.1925	1
Total P	0.0454*	0.5418	0.6806
Ca^{2+}	0.1529	0.4032	1
K^+	0.0012**	0.7736	0.0187*
Mg^{2+}	0.7198	-0.1054	1
Na^{2+}	0.4727	0.2093	1
Si	0.0016**	0.7596	0.0260*
Cl^-	0.1170	-0.4383	1
SO_2^{4-}	0.5183	0.1887	1

Table S3: Actual diatom valve area compared with estimates of valve area using linear measurements and shape approximations, for diatom species with an unusual valve shape. Thirteen species showed an unusual valve shape compared to that described for their genus in Hillebrand *et al* (1999). For twelve of these species the new shape approximation was on average a better estimate of the actual valve area than when using the original shape approximation. For one species (*Eunotia bilunaris*) it was not possible to use width and length measurements to calculate the area using the original shape approximation, but the new area shape approximation is a good estimate of the actual area for that species.

**Species**	**Stream**	**Actual valve area (μm^2)**	**Width (μm)**	**Length (μm)**	**Original volume shape approximation**	**Original area shape approximation**	**Original estimated area**	**New area shape approximation**	**New estimated area**	**Best shape approximation**
Eunotia bilunaris	b13	51.26	3.33	16.10	Sickle shaped prism	-	-	Rectangle	53.53	-
Eunotia bilunaris	m3	49.27	2.66	22.26	Sickle shaped prism	-	-	Rectangle	59.14	-
Gomphonema angustatum	i9	71.74	5.35	17.81	Gomphonemoid	Petal	51.78	Ellipse	74.90	New
Gomphonema clavatum	c7	105.77	5.18	24.88	Gomphonemoid	Petal	68.30	Ellipse	101.27	New
Gomphonema clavatum	m3	233.87	8.50	35.36	Gomphonemoid	Petal	256.66	Ellipse	236.03	New
Gomphonema clavatum	e4	139.35	6.57	28.35	Gomphonemoid	Petal	160.10	Ellipse	146.34	New
Gomphonema clavatum	d14	96.17	5.70	20.89	Gomphonemoid	Petal	99.39	Ellipse	93.57	New
Gomphonema clevei	c7	175.36	6.14	33.36	Gomphonemoid	Petal	107.78	Ellipse	160.85	New
Gomphonema clevei	i9	241.87	6.82	46.86	Gomphonemoid	Petal	166.31	Ellipse	250.82	New
Gomphonema clevei	j6	210.66	6.56	39.85	Gomphonemoid	Petal	235.28	Ellipse	205.32	New
Gomphonema clevei	g16	175.59	6.43	38.44	Gomphonemoid	Petal	222.12	Ellipse	194.19	New
Gomphonema parvulum	n8	92.26	5.67	24.43	Gomphonemoid	Petal	73.81	Ellipse	108.75	New
Gomphonema parvulum	k5	28.23	3.65	9.92	Gomphonemoid	Petal	20.01	Ellipse	28.45	New
Gomphonema parvulum	l1	65.53	5.16	18.22	Gomphonemoid	Petal	50.77	Ellipse	73.79	New
Gomphonema parvulum	j6	42.86	4.22	13.88	Gomphonemoid	Petal	47.76	Ellipse	46.00	New
Gomphonema parvulum	h12	68.54	5.02	18.18	Gomphonemoid	Petal	75.97	Ellipse	71.69	New
Gomphonema parvulum	g16	91.36	6.03	19.70	Gomphonemoid	Petal	96.79	Ellipse	93.39	New
Gomphonema type D	c7	32.33	3.59	10.67	Gomphonemoid	Petal	21.03	Ellipse	30.13	New
Gomphonema type D	i9	28.44	3.32	10.45	Gomphonemoid	Petal	18.91	Ellipse	27.24	New
Gomphonema type D	m3	36.86	4.40	12.84	Gomphonemoid	Petal	44.73	Ellipse	44.37	New
Gomphonema type D	e4	42.28	3.90	12.64	Gomphonemoid	Petal	40.03	Ellipse	38.68	Old
Gomphonema type D	j6	33.98	3.64	10.93	Gomphonemoid	Petal	31.73	Ellipse	31.24	Old
Gomphonema type D	h12	46.07	4.44	13.12	Gomphonemoid	Petal	46.26	Ellipse	45.73	Old
Gomphonema type D	d14	28.47	3.29	10.69	Gomphonemoid	Petal	28.57	Ellipse	27.59	Old
Gomphonema type D	g16	31.30	3.71	10.47	Gomphonemoid	Petal	30.50	Ellipse	30.53	New
Meridion circulare	m3	109.46	5.47	26.48	Gomphonemoid	Petal	126.77	Ellipse	113.86	New
Meridion circulare	e4	125.38	5.21	32.75	Gomphonemoid	Petal	154.08	Ellipse	133.95	New
Meridion circulare	a10	128.90	5.56	29.37	Gomphonemoid	Petal	144.56	Ellipse	128.29	New
Meridion circulare	h12	74.32	4.27	23.28	Gomphonemoid	Petal	88.32	Ellipse	78.05	New
Meridion circulare	d14	144.87	4.09	43.37	Gomphonemoid	Petal	167.07	Ellipse	139.18	New

Meridion circulare	g16	69.02	5.42	17.73	Gomphonemoid	Petal	78.33	Ellipse	75.55	New
Nitzschia dissipata	b13	93.26	4.60	27.57	Prism on parallelogram	Parallelogram	126.90	Ellipse	99.67	New
Nitzschia dissipata	e4	18.68	2.91	10.45	Prism on parallelogram	Parallelogram	30.39	Ellipse	23.87	New
Nitzschia dissipata	h12	77.34	3.78	25.51	Prism on parallelogram	Parallelogram	96.37	Ellipse	75.69	New
Nitzschia dissipata	d14	81.28	2.99	30.92	Prism on parallelogram	Parallelogram	92.58	Ellipse	72.71	New
Nitzschia dissipata	g16	64.44	3.40	24.48	Prism on parallelogram	Parallelogram	83.26	Ellipse	65.39	New
Nitzschia fonticola	e4	65.63	3.72	24.28	Prism on parallelogram	Parallelogram	90.21	Ellipse	70.85	New
Nitzschia fonticola	h12	34.54	2.61	16.29	Prism on parallelogram	Parallelogram	42.51	Ellipse	33.39	New
Nitzschia inconspicua	c7	13.71	2.76	6.23	Prism on parallelogram	Parallelogram	17.21	Ellipse	13.52	New
Nitzschia inconspicua	d14	14.63	2.70	7.20	Prism on parallelogram	Parallelogram	19.47	Ellipse	15.29	New
Nitzschia inconspicua	g16	20.55	2.54	9.11	Prism on parallelogram	Parallelogram	23.09	Ellipse	18.14	New
Nitzschia palea	l1	36.45	2.36	20.46	Prism on parallelogram	Parallelogram	48.37	Ellipse	37.99	New
Nitzschia palea	f11	33.30	2.38	17.08	Prism on parallelogram	Parallelogram	40.69	Ellipse	31.96	New
Nitzschia palea	m3	73.94	3.17	28.32	Prism on parallelogram	Parallelogram	89.78	Ellipse	70.51	New
Nitzschia palea	e4	47.67	2.60	25.91	Prism on parallelogram	Parallelogram	67.29	Ellipse	52.85	New
Nitzschia palea	j6	38.58	2.57	19.07	Prism on parallelogram	Parallelogram	49.09	Ellipse	38.55	New
Nitzschia palea	h12	120.40	3.66	37.55	Prism on parallelogram	Parallelogram	137.44	Ellipse	107.95	New
Nitzschia palea	d14	52.29	2.42	25.70	Prism on parallelogram	Parallelogram	62.17	Ellipse	48.83	New
Nitzschia palea	g16	45.95	2.54	23.78	Prism on parallelogram	Parallelogram	60.31	Ellipse	47.37	New
Nitzschia paleacea	k5	55.25	2.73	26.82	Prism on parallelogram	Parallelogram	73.25	Ellipse	57.53	New
Nitzschia paleacea	f11	36.61	2.13	20.94	Prism on parallelogram	Parallelogram	44.53	Ellipse	34.97	New
Nitzschia paleacea	l1	28.27	1.95	16.93	Prism on parallelogram	Parallelogram	33.06	Ellipse	25.97	New
Nitzschia paleacea	i9	27.73	2.73	14.19	Prism on parallelogram	Parallelogram	38.78	Ellipse	30.46	New
Nitzschia paleacea	m3	77.27	2.61	37.21	Prism on parallelogram	Parallelogram	97.27	Ellipse	76.39	New
Nitzschia paleacea	e4	64.57	2.15	35.34	Prism on parallelogram	Parallelogram	66.48	Ellipse	59.59	Old
Nitzschia paleacea	h12	32.64	1.96	22.75	Prism on parallelogram	Parallelogram	44.49	Ellipse	34.94	New
Nitzschia paleacea	d14	39.48	1.84	25.32	Prism on parallelogram	Parallelogram	46.63	Ellipse	36.62	New
Nitzschia paleacea	g16	50.46	2.24	29.30	Prism on parallelogram	Parallelogram	65.68	Ellipse	51.58	New
Rhoicosphenia abbreviata	n8	40.01	3.43	14.50	Gomphonemoid	Petal	26.51	Ellipse	39.01	New
Rhoicosphenia abbreviata	i9	106.48	4.98	26.95	Gomphonemoid	Petal	70.60	Ellipse	105.35	New
Rhoicosphenia abbreviata	k5	77.68	4.37	22.38	Gomphonemoid	Petal	51.60	Ellipse	76.77	New
Rhoicosphenia abbreviata	l1	49.28	3.21	20.41	Gomphonemoid	Petal	34.22	Ellipse	51.45	New
Rhoicosphenia abbreviata	m3	67.30	4.86	19.54	Gomphonemoid	Petal	80.67	Ellipse	74.64	New

Table S4: Slopes and p-values from linear regressions of temperature against log_{10} valve width for 31 species of diatom found in at least 2 of the sampled streams. Corrected p-values are Bonferroni corrected and stars represent levels of significance: p<0.05 (*), p<0.01 (**), p<0.001 (***).

Species	num.streams	Slope	R^2	p-value	Corrected p-value
Achnanthes lanceolata	13	-0.0008	0.0088	0.2947	1
Amphora inariensis	6	-0.0021	0.0303	0.1957	1
Amphora pediculus	6	-0.001	0.0153	0.3456	1
Caloneis lauta	2	0.0036	0.0739	0.2464	1
Cocconeis pediculus	4	0.0204	0.0923	0.0760	1
Cocconeis placentula	10	-0.0006	0.0013	0.7169	1
Cymbella sinuata	2	0.0155	0.5588	0.0002***	0.0047**
Diatoma mesodon	7	-0.0017	0.0188	0.2578	1
Epithemia sorex	3	-0.0057	0.069	0.1770	1
Epithemia turgida	8	0.0004	0.0003	0.8836	1
Fragilaria arcus	2	0.0035	0.1931	0.0526	1
Fragilaria capucina	14	0.0022	0.0269	0.0527	1
Fragilaria construens	2	0.0102	0.0432	0.4076	1
Fragilaria pinnata	8	-0.0004	0.0022	0.7077	1
Gomphonema clavatum	4	0.0058	0.2936	0.0011**	0.0350*
Gomphonema clevei	4	0.001	0.0178	0.4598	1
Gomphonema parvulum	6	-0.0555	0.524	0.00000028***	0.0000087***
Gomphonema type D	8	0.0039	0.1473	0.0010**	0.0322*
Melosira varians	8	0.0171	0.1896	0.0001***	0.0029**
Meridion circulare	10	0	0	0.9866	1
Navicula atomus	4	0.0001	0.0001	0.9531	1
Navicula minima	13	0.0013	0.0236	0.0872	1
Navicula placentula	6	-0.0037	0.2454	0.0001***	0.0028**
Nitzschia dissipata	4	0.002	0.0046	0.7036	1
Nitzschia fonticola	2	-0.0227	0.0553	0.3634	1
Nitzschia inconspicua	3	0.0004	0.0009	0.9049	1
Nitzschia palea	8	0.0008	0.0035	0.6027	1
Nitzschia paleacea	9	-0.0013	0.0067	0.4609	1
Rhoicosphenia abbreviata	5	-0.0118	0.2238	0.0009***	0.0279*
Rhopalodia gibba	5	-0.0032	0.0484	0.1288	1
Synedra ulna	10	-0.0009	0.0081	0.4177	1

Table S5: Matrix showing the proportion of the total community size change between streams that is due to intraspecific effects. Each value is a pairwise comparison between streams α and β, calculating the size change we would expect to see due to intraspecific effects as a percentage of the overall average community size difference between the streams, $\Delta_{\alpha\beta}^{INTRA}/\Delta$

The matrix shows only upper triangle values as the matrix is symmetric.

	a	b	c	d	e	f	g	h	i	j	k	l	m	n
a	-	0.1246	0.6249	-0.5721	1.7914	0.1537	-16.8708	0.2912	-0.0617	-0.6561	-0.2373	-2.4133	0.0832	0.1145
b	-	-	-0.9684	-0.1268	-0.3433	0.0028	-0.0672	0.1031	-0.0125	-0.1481	0.0982	-0.1388	-0.4554	0.0207
c	-	-	-	0.1611	0.3826	0.2600	0.3198	3.0254	0.0927	-0.0522	0.0601	0.2787	-1.9884	-0.0129
d	-	-	-	-	0.0868	0.6912	-0.1634	-0.1020	0.1973	-0.4639	-0.0368	0.5485	0.0127	-1.1347
e	-	-	-	-	-	0.2665	0.0232	-0.3243	0.0118	-0.2643	0.0608	-0.9984	-0.3046	-0.3390
f	-	-	-	-	-	-	0.0608	0.0395	-0.5920	0.4849	0.1443	0.4064	0.0049	1.5522
g	-	-	-	-	-	-	-	-0.5074	-0.0836	-0.1543	-0.5409	-2.3370	-0.3259	-0.5350
h	-	-	-	-	-	-	-	-	-0.0746	-0.2587	-0.0251	-0.4289	-0.0941	-0.0116
i	-	-	-	-	-	-	-	-	-	-0.0455	-0.0177	0.0650	0.0041	0.8441
j	-	-	-	-	-	-	-	-	-	-	0.7168	-0.5082	-0.2757	-0.2906
k	-	-	-	-	-	-	-	-	-	-	-	1.4167	-0.1817	-0.7884
l	-	-	-	-	-	-	-	-	-	-	-	-	-0.3994	-0.6093
m	-	-	-	-	-	-	-	-	-	-	-	-	-	-0.3872
n	-	-	-	-	-	-	-	-	-	-	-	-	-	-

Table S6: Matrix showing the total relative abundance of species in stream α (labelled in the rows) not found in stream β (labelled in the columns; see Methods). Each value represents the proportional contribution of species turnover to overall species compositional differences between streams.

	a	b	c	d	e	f	g	h	i	j	k	l	m	n
a	0	0.0417	0.0254	0.0048	0.0060	0.0091	0.0054	0.0284	0.0260	0.2958	0.0387	0.0526	0.0296	0.0405
b	0.0334	0	0.0409	0.1438	0.1378	0.0238	0.0349	0.1478	0.0423	0.3302	0.0371	0.1493	0.1568	0.0509
c	0.0332	0.4097	0	0.0541	0.0897	0.0356	0.0360	0.1458	0.0288	0.2219	0.2711	0.3100	0.0973	0.2439
d	0.1061	0.1700	0.0662	0	0.0423	0.0646	0.0591	0.0343	0.0271	0.4773	0.0838	0.0678	0.0726	0.1429
e	0.1236	0.2712	0.1525	0.0598	0	0.1342	0.0676	0.0714	0.1380	0.3909	0.2181	0.1477	0.0907	0.3156
f	0.1208	0.3343	0.0449	0.0266	0.0256	0	0.0118	0.3028	0.0123	0.1203	0.0227	0.0232	0.0863	0.0700
g	0.1107	0.2611	0.1144	0.0351	0.0443	0.1042	0	0.1125	0.1181	0.2740	0.1393	0.1439	0.0913	0.2122
h	0.1296	0.2438	0.0926	0.0504	0.0576	0.0998	0.0484	0	0.1255	0.2407	0.2253	0.0751	0.0936	0.2994
i	0.1426	0.7166	0.1442	0.0561	0.4365	0.0418	0.0865	0.2037	0	0.0856	0.1156	0.3986	0.4378	0.4964
j	0.0960	0.5144	0.0736	0.0252	0.2283	0.0438	0.0196	0.1463	0.0457	0	0.1463	0.1966	0.2022	0.4651
k	0.3592	0.2836	0.2033	0.2602	0.3365	0.2428	0.1819	0.2716	0.2555	0.0963	0	0.0649	0.0883	0.1940
l	0.5626	0.2645	0.2251	0.1937	0.2367	0.0824	0.0731	0.1995	0.0916	0.3248	0.0371	0	0.1067	0.2448
m	0.1090	0.2042	0.0673	0.0267	0.0557	0.0638	0.0731	0.1079	0.0487	0.0870	0.0313	0.0487	0	0.0893
n	0.6897	0.6405	0.0596	0.0460	0.5559	0.0669	0.0115	0.1567	0.0627	0.0157	0.0052	0.0199	0.2623	0

Table S7: Intraspecific analysis for abiotic variables pH, Si, K, NO_3, NH_4, Total N, PO_4 and Total P. Slopes and p-values are from linear regressions of the abiotic variable against mean log_{10} valve area, for 31 species of diatoms found in at least 2 of the sampled streams. Corrected p-values are Bonferroni corrected and stars represent levels of significance: p<0.05 (*), p<0.01 (**), p<0.001 (***).

Abiotic variable	Species	num.streams	Slope	R^2	p-value	Corrected p-value
pH	*Achnanthes lanceolata*	13	-0.0018	0	0.9794	1
	Amphora inariensis	6	-0.3956	0.1979	0.00053***	0.0157593*
	Amphora pediculus	6	-0.1366	0.0758	0.0332*	0.9974817
	Caloneis lauta	2	0.1597	0.0123	0.6410	1
	Cocconeis pediculus	4	-0.4741	0.1075	0.0545	1
	Cocconeis placentula	10	-0.1708	0.0207	0.1528	1
	Cymbella sinuata	2	1.8849	0.5866	0.000083***	0.0024798**
	Diatoma mesodon	7	-0.1668	0.0642	0.0343*	1
	Epithemia sorex	3	0.2295	0.1404	0.0495*	1
	Epithemia turgida	8	0.8731	0.258	0.000006***	0.0001842***
	Fragilaria arcus	2	NA	0	NA	NA
	Fragilaria capucina	14	0.0318	0.0026	0.5481	1
	Fragilaria construens	2	0.3043	0.097	0.2083	1
	Fragilaria pinnata	8	-0.024	0.0009	0.8124	1
	Gomphonema clavatum	4	0.2695	0.1476	0.0273*	0.8179845
	Gomphonema clevei	4	0.1953	0.1584	0.0218*	0.6545091
	Gomphonema parvulum	6	-0.1906	0.0147	0.4679	1
	Gomphonema type D	8	-0.01	0.0005	0.8534	1
	Melosira varians	8	0.5615	0.0375	0.0959	1
	Meridion circulare	10	0.036	0.0015	0.7183	1
	Navicula atomus	4	-0.0576	0.0067	0.6154	1
	Navicula minima	13	-0.0885	0.0231	0.0910	1
	Navicula placentula	6	0.1025	0.0154	0.3571	1
	Nitzschia dissipata	4	0.2414	0.0368	0.2773	1
	Nitzschia fonticola	2	-0.6418	0.0645	0.3255	1
	Nitzschia inconspicua	3	0.2217	0.1549	0.1061	1
	Nitzschia palea	8	-0.435	0.1261	0.0013**	0.0396801*
	Nitzschia paleacea	9	-0.364	0.0887	0.0062**	0.1874952
	Rhoicosphenia abbreviata	5	0.0034	0	0.9871	1
	Rhopalodia gibba	5	0.0207	0.0002	0.9187	1
	Synedra ulna	10	0.1164	0.0119	0.3257	1
Si	*Achnanthes lanceolata*	13	0.0001	0	0.9808	1
	Amphora inariensis	6	-0.0136	0.2609	0.000049***	0.0015221**
	Amphora pediculus	6	-0.0044	0.0681	0.0439*	1
	Caloneis lauta	2	0.0033	0.0123	0.6410	1
	Cocconeis pediculus	4	-0.0568	0.0224	0.3911	1
	Cocconeis placentula	10	0.0025	0.0052	0.4743	1
	Cymbella sinuata	2	0.0857	0.5866	0.000083***	0.00256246**
	Diatoma mesodon	7	-0.0066	0.039	0.1011	1
	Epithemia sorex	3	0.0193	0.0346	0.3430	1
	Epithemia turgida	8	0.0218	0.174	0.0003***	0.00918716**
	Fragilaria arcus	2	0.0335	0.6105	0.000048***	0.00147405**
	Fragilaria capucina	14	-0.0009	0.0019	0.6056	1

	Fragilaria construens	2	0.0103	0.097	0.2083	1
	Fragilaria pinnata	8	0.0007	0.0005	0.8532	1
	Gomphonema clavatum	4	0.0178	0.4045	0.00007***	0.00215481**
	Gomphonema clevei	4	0.0069	0.1115	0.0575	1
	Gomphonema parvulum	6	-0.0507	0.1153	0.0370	1
	Gomphonema type D	8	0.002	0.0123	0.3601	1
	Melosira varians	8	0.0522	0.3993	<2.2e-16***	<2.2e-16***
	Meridion circulare	10	-0.0002	0.0001	0.9346	1
	Navicula atomus	4	-0.0026	0.0205	0.3785	1
	Navicula minima	13	0.001	0.0032	0.5295	1
	Navicula placentula	6	-0.0069	0.0623	0.0612	1
	Nitzschia dissipata	4	0.0183	0.1035	0.0636	1
	Nitzschia fonticola	2	-0.0165	0.0645	0.3255	1
	Nitzschia inconspicua	3	0.0112	0.1874	0.0728	1
	Nitzschia palea	8	0.0035	0.0106	0.3670	1
	Nitzschia paleacea	9	-0.0095	0.0607	0.0248*	0.76752838
	Rhoicosphenia abbreviata	5	-0.0724	0.176	0.0037**	0.11475425
	Rhopalodia gibba	5	-0.0026	0.0087	0.5235	1
	Synedra ulna	10	0.0005	0.0003	0.8848	1
K	*Achnanthes lanceolata*	13	-0.0001	0	0.9964	1
	Amphora inariensis	6	-0.1064	0.2571	0.00006***	0.00176669**
	Amphora pediculus	6	-0.0411	0.1125	0.0088**	0.27292772
	Caloneis lauta	2	0.0213	0.0123	0.6410	1
	Cocconeis pediculus	4	-0.13	0.0647	0.1404	1
	Cocconeis placentula	10	-0.0176	0.0046	0.5026	1
	Cymbella sinuata	2	0.2356	0.5866	0.000083***	0.00256246**
	Diatoma mesodon	7	-0.046	0.0209	0.2325	1
	Epithemia sorex	3	0.0865	0.1402	0.0496*	1
	Epithemia turgida	8	0.1675	0.2429	0.000013***	0.00039246***
	Fragilaria arcus	2	0.2179	0.6105	0.000048***	0.00147405**
	Fragilaria capucina	14	-0.0013	0.0001	0.9099	1
	Fragilaria construens	2	0.0761	0.097	0.2083	1
	Fragilaria pinnata	8	0.0008	0	0.9753	1
	Gomphonema clavatum	4	0.1275	0.304	0.00088***	0.02733487*
	Gomphonema clevei	4	0.0373	0.0513	0.2050	1
	Gomphonema parvulum	6	-0.076	0.0511	0.1724	1
	Gomphonema type D	8	0	0	0.9979	1
	Melosira varians	8	0.3275	0.3716	0.00000001***	0.00000031***
	Meridion circulare	10	0.0121	0.0029	0.6117	1
	Navicula atomus	4	-0.0277	0.027	0.3108	1
	Navicula minima	13	-0.001	0.0001	0.9294	1
	Navicula placentula	6	-0.0441	0.0524	0.0869	1
	Nitzschia dissipata	4	0.1278	0.1011	0.0668	1
	Nitzschia fonticola	2	-0.1167	0.0645	0.3255	1
	Nitzschia inconspicua	3	0.1394	0.1459	0.1178	1
	Nitzschia palea	8	0.0123	0.0031	0.6271	1
	Nitzschia paleacea	9	-0.0473	0.038	0.0775	1
	Rhoicosphenia abbreviata	5	-0.141	0.1751	0.0038**	0.11790943
	Rhopalodia gibba	5	-0.011	0.003	0.7068	1
	Synedra ulna	10	-0.0226	0.01	0.3687	1

NO_3	*Achnanthes lanceolata*	13	-0.2492	0	0.9728	1
	Amphora inariensis	6	-16.0934	0.0325	0.1798	1
	Amphora pediculus	6	-9.4507	0.04	0.1257	1
	Caloneis lauta	2	-15.9656	0.0123	0.6410	1
	Cocconeis pediculus	4	17.675	0.0339	0.2896	1
	Cocconeis placentula	10	-27.0861	0.056	0.0178*	0.55152565
	Cymbella sinuata	2	188.4893	0.5866	0.000083***	0.00256246**
	Diatoma mesodon	7	-19.0916	0.055	0.0507	1
	Epithemia sorex	3	28.0167	0.1834	0.0230*	0.71247889
	Epithemia turgida	8	-52.9087	0.0929	0.0098**	0.30266044
	Fragilaria arcus	2	217.9108	0.6105	0.000048***	0.00147405**
	Fragilaria capucina	14	7.7439	0.0157	0.1406	1
	Fragilaria construens	2	-20.2895	0.097	0.2083	1
	Fragilaria pinnata	8	8.5085	0.0127	0.3670	1
	Gomphonema clavatum	4	42.1836	0.2073	0.0078**	0.24032037
	Gomphonema clevei	4	46.1146	0.2531	0.0028**	0.08815222
	Gomphonema parvulum	6	-16.5008	0.0156	0.4546	1
	Gomphonema type D	8	20.9695	0.1597	0.0006***	0.01893108*
	Melosira varians	8	-80.7667	0.1095	0.0037**	0.1158
	Meridion circulare	10	-32.1535	0.1193	0.0007***	0.02307392*
	Navicula atomus	4	1.9582	0.0028	0.7468	1
	Navicula minima	13	-4.9876	0.0084	0.3081	1
	Navicula placentula	6	24.7112	0.0792	0.0340*	1
	Nitzschia dissipata	4	-23.4619	0.0267	0.3555	1
	Nitzschia fonticola	2	128.3546	0.0645	0.3255	1
	Nitzschia inconspicua	3	45.2029	0.1895	0.0710	1
	Nitzschia palea	8	35.3918	0.1105	0.0028**	0.08579002
	Nitzschia paleacea	9	31.2836	0.0774	0.0109*	0.33690645
	Rhoicosphenia abbreviata	5	-35.5407	0.076	0.0638	1
	Rhopalodia gibba	5	23.1284	0.0821	0.0460*	1
	Synedra ulna	10	4.5006	0.0021	0.6787	1
NH_4	*Achnanthes lanceolata*	13	-3.1618	0.0058	0.3963	1
	Amphora inariensis	6	17.7961	0.1535	0.0026**	0.07992141
	Amphora pediculus	6	4.1558	0.0216	0.2630	1
	Caloneis lauta	2	6.3862	0.0123	0.6410	1
	Cocconeis pediculus	4	0.5334	0.0001	0.9553	1
	Cocconeis placentula	10	24.9675	0.1163	0.0005***	0.01597616*
	Cymbella sinuata	2	-31.4149	0.5866	0.000083***	0.00256246**
	Diatoma mesodon	7	3.8226	0.0128	0.3512	1
	Epithemia sorex	3	-28.0167	0.1834	0.0230*	0.71247889
	Epithemia turgida	8	0.5655	0	0.9742	1
	Fragilaria arcus	2	-108.9554	0.6105	0.000048***	0.00147405**
	Fragilaria capucina	14	-2.8964	0.007	0.3249	1
	Fragilaria construens	2	8.6955	0.097	0.2083	1
	Fragilaria pinnata	8	-5.9214	0.0336	0.1409	1
	Gomphonema clavatum	4	44.4309	0.2526	0.0029**	0.08904037
	Gomphonema clevei	4	-21.7557	0.1462	0.0281*	0.87122741
	Gomphonema parvulum	6	46.2228	0.3497	0.000092***	0.00286471**
	Gomphonema type D	8	2.0408	0.0027	0.6699	1
	Melosira varians	8	-35.0476	0.0341	0.1127	1

	Meridion circulare	10	1.673	0.0014	0.7250	1
	Navicula atomus	4	-2.3772	0.0202	0.3814	1
	Navicula minima	13	5.0587	0.027	0.0670	1
	Navicula placentula	6	4.9552	0.0128	0.4015	1
	Nitzschia dissipata	4	22.0418	0.0806	0.1038	1
	Nitzschia fonticola	2	-32.0886	0.0645	0.3255	1
	Nitzschia inconspicua	3	16.7255	0.1459	0.1178	1
	Nitzschia palea	8	-4.4693	0.0037	0.5962	1
	Nitzschia paleacea	9	15.7363	0.0705	0.0152*	0.47273109
	Rhoicosphenia abbreviata	5	-0.6197	0.0001	0.9478	1
	Rhopalodia gibba	5	-5.62	0.01	0.4946	1
	Synedra ulna	10	-10.0702	0.0187	0.2176	1
Total N	*Achnanthes lanceolata*	13	0.2533	0.0042	0.4708	1
	Amphora inariensis	6	0.6072	0.0191	0.3058	1
	Amphora pediculus	6	0.4294	0.0229	0.2480	1
	Caloneis lauta	2	-1.4514	0.0123	0.6410	1
	Cocconeis pediculus	4	1.6636	0.0292	0.3264	1
	Cocconeis placentula	10	1.5837	0.0391	0.0486*	1
	Cymbella sinuata	2	-2.8559	0.5866	0.000083***	0.00256246**
	Diatoma mesodon	7	0.3103	0.0078	0.4674	1
	Epithemia sorex	3	-2.6829	0.2079	0.0148*	0.45747103
	Epithemia turgida	8	-1.6702	0.0537	0.0517	1
	Fragilaria arcus	2	-2.5047	0.6105	0.000048***	0.00147405**
	Fragilaria capucina	14	-0.3434	0.0126	0.1865	1
	Fragilaria construens	2	2.2544	0.097	0.2083	1
	Fragilaria pinnata	8	-0.5438	0.0176	0.2887	1
	Gomphonema clavatum	4	0.1067	0.0013	0.8398	1
	Gomphonema clevei	4	1.2914	0.0383	0.2749	1
	Gomphonema parvulum	6	2.0172	0.1117	0.0402*	1
	Gomphonema type D	8	0.5144	0.059	0.0427*	1
	Melosira varians	8	-2.7063	0.0551	0.0427*	1
	Meridion circulare	10	0.8674	0.0362	0.0693	1
	Navicula atomus	4	-0.4734	0.0347	0.2498	1
	Navicula minima	13	0.1313	0.0023	0.5942	1
	Navicula placentula	6	1.0006	0.114	0.0102*	0.31697779
	Nitzschia dissipata	4	1.2899	0.0488	0.2093	1
	Nitzschia fonticola	2	-2.2518	0.0645	0.3255	1
	Nitzschia inconspicua	3	1.0016	0.1369	0.1307	1
	Nitzschia palea	8	-0.5983	0.0176	0.2439	1
	Nitzschia paleacea	9	-0.2718	0.0033	0.6060	1
	Rhoicosphenia abbreviata	5	1.5696	0.0251	0.2930	1
	Rhopalodia gibba	5	1.1985	0.0278	0.2520	1
	Synedra ulna	10	0.9837	0.0397	0.0710	1
PO_4	*Achnanthes lanceolata*	13	0.6854	0.0007	0.7714	1
	Amphora inariensis	6	2.2997	0.0034	0.6686	1
	Amphora pediculus	6	-1.4239	0.0078	0.5020	1
	Caloneis lauta	2	3.9914	0.0123	0.6410	1
	Cocconeis pediculus	4	-1.1374	0.0005	0.8988	1
	Cocconeis placentula	10	-8.6991	0.0531	0.0211*	0.6333786
	Cymbella sinuata	2	188.4893	0.5866	0.000083***	0.0024798**

	Diatoma mesodon	7	-1.0984	0.0027	0.6703	1
	Epithemia sorex	3	1.0564	0.0099	0.6153	1
	Epithemia turgida	8	18.6956	0.1789	0.00024***	0.0071391**
	Fragilaria arcus	2	12.8183	0.6105	0.000048***	0.0014265**
	Fragilaria capucina	14	-0.5672	0.0007	0.7490	1
	Fragilaria construens	2	30.4343	0.097	0.2083	1
	Fragilaria pinnata	8	6.9597	0.0242	0.2124	1
	Gomphonema clavatum	4	-3.9036	0.0174	0.4638	1
	Gomphonema clevei	4	8.8223	0.1633	0.0197*	0.5899428
	Gomphonema parvulum	6	8.042	0.037	0.2475	1
	Gomphonema type D	8	-4.4506	0.107	0.0057**	0.1716
	Melosira varians	8	17.8197	0.0694	0.0224*	0.6719
	Meridion circulare	10	0.2763	0.0001	0.9458	1
	Navicula atomus	4	1.4114	0.0052	0.6598	1
	Navicula minima	13	-2.7757	0.0141	0.1873	1
	Navicula placentula	6	-14.2698	0.1502	0.0029**	0.0867693
	Nitzschia dissipata	4	21.9905	0.0084	0.6056	1
	Nitzschia fonticola	2	NA	0	NA	NA
	Nitzschia inconspicua	3	-9.2132	0.1802	0.0791	1
	Nitzschia palea	8	5.4968	0.0101	0.3789	1
	Nitzschia paleacea	9	-7.6717	0.0733	0.0133*	0.3994572
	Rhoicosphenia abbreviata	5	4.4544	0.0314	0.2388	1
	Rhopalodia gibba	5	-80.7322	0.3069	0.000036***	0.0010914**
	Synedra ulna	10	-5.7454	0.0394	0.0722	1
Total P	*Achnanthes lanceolata*	13	-0.3412	0.0004	0.8164	1
	Amphora inariensis	6	-4.6539	0.0451	0.1129	1
	Amphora pediculus	6	-1.923	0.0322	0.1701	1
	Caloneis lauta	2	1.5966	0.0123	0.6410	1
	Cocconeis pediculus	4	-3.8733	0.0187	0.4332	1
	Cocconeis placentula	10	-5.3115	0.0336	0.0678	1
	Cymbella sinuata	2	23.5612	0.5866	0.000083***	0.00256246**
	Diatoma mesodon	7	-1.633	0.0146	0.3193	1
	Epithemia sorex	3	1.6728	0.0393	0.3120	1
	Epithemia turgida	8	13.2589	0.2562	0.000007***	0.0002077***
	Fragilaria arcus	2	8.3812	0.6105	0.000048***	0.00147405**
	Fragilaria capucina	14	0.2943	0.0006	0.7832	1
	Fragilaria construens	2	5.0724	0.097	0.2083	1
	Fragilaria pinnata	8	2.3508	0.0081	0.4718	1
	Gomphonema clavatum	4	1.9995	0.014	0.5113	1
	Gomphonema clevei	4	11.2352	0.2829	0.0014**	0.04479314*
	Gomphonema parvulum	6	-0.7892	0.001	0.8483	1
	Gomphonema type D	8	-2.8145	0.1117	0.0047**	0.1453
	Melosira varians	8	17.5301	0.1672	0.00027***	0.00835853**
	Meridion circulare	10	-0.9873	0.0015	0.7171	1
	Navicula atomus	4	0.719	0.0018	0.7937	1
	Navicula minima	13	-1.4375	0.012	0.2232	1
	Navicula placentula	6	-5.0093	0.1094	0.0120*	0.37054827
	Nitzschia dissipata	4	8.2189	0.0331	0.3028	1
	Nitzschia fonticola	2	-42.7849	0.0645	0.3255	1
	Nitzschia inconspicua	3	-6.7049	0.0938	0.2165	1

Nitzschia palea	8	-0.6939	0.001	0.7812	1
Nitzschia paleacea	9	-4.0237	0.0516	0.0389*	1
Rhoicosphenia abbreviata	5	3.0752	0.0232	0.3125	1
Rhopalodia gibba	5	-15.1778	0.1839	0.0021**	0.06544503
Synedra ulna	10	-3.8554	0.0333	0.0989	1

Table S8: Summary of regression slopes from Table 2 and Table S7, showing the total number of positive and negative slopes, positive and negative slopes with $p < 0.05$ and positive and negative slopes with Bonferroni-corrected $p < 0.05$

	**All slopes**		**Significant slopes**		**Bonferroni corrected**	
**Abiotic variable**	**Positive**	**Negative**	**Positive**	**Negative**	**Positive**	**Negative**
Temperature	15	16	5	7	4	3
pH	16	14	6	5	2	2
Si	18	13	5	4	5	1
K	15	16	6	3	5	1
NO_3	17	14	10	4	3	1
NH_4	17	14	5	4	2	2
Total N	19	12	4	4	0	2
PO_4	17	13	5	5	3	1
Total P	14	17	5	4	5	0

Table S9: Summary of p-values from linear and quadratic regressions of log_{10} mean valve area against 9 different abiotic variables. Corrected p-values are Bonferroni corrected and stars represent levels of significance: p<0.05 (*), p<0.01 (**), p<0.001 (***).

	Linear regression				Quadratic regression		
**Abiotic variable**	**Slope**	**R^2**	**p-value**	**Corrected p-value**	**R^2**	**p-value**	**Corrected p-value**
Temperature	0.0058	0.1032	0.2628	1	0.1619	0.3785	1
pH	0.4484	0.5759	0.0016**	0.0148*	0.6002	0.0065**	0.0581
Si	0.0097	0.2523	0.0672	0.6048	0.2680	0.1799	1
K	0.0659	0.2295	0.0830	0.7473	0.2445	0.2139	1
NO_3	4.0969	0.0045	0.8195	1	0.0701	0.6707	1
NH_4	-16.4759	0.2181	0.0923	0.8303	0.2533	0.2006	1
Total N	-0.1710	0.0034	0.8439	1	0.0206	0.8920	1
PO_4	6.0221	0.0835	0.3165	1	0.2702	0.1768	1
Total P	5.4506	0.1874	0.1221	1	0.2333	0.2320	1

Table S12: Species found in the fourteen streams that were used for analysis, listed with their species authorities. Two taxa that were sampled could not be identified to species level. The first we called Gomphonema Type D; it is very likely that it belongs in the Gomphonema genus and is distinct from other Gomphonema species in the streams. The second we called Pinnularia spp.; it is very likely that it belongs in the Pinnularia genus, and it was only found in one stream.

**Species**	**Authority**
Achnanthes grana	Hohn & Hellermann
Achnanthes lanceolata	(Brébissson) Grunow
Achnanthes minutissima	Kützing
Achnanthes stolida	(Krasske) Krasske
Amphora inariensis	Krammer
Amphora ovalis	(Kützing) Kützing
Amphora pediculus	(Kützing) Grunow
Caloneis lauta	Carter & Bailey-Watts
Cocconeis pediculus	Ehrenberg
Cocconeis placentula	Ehrenberg
Cymbella minuta	Hilse
Cymbella sinuata	Gregory
Diatoma mesodon	(Ehrenberg) Kützing
Diploneis ovalis	Hilse (Cleve)
Epithemia sorex	Kützing
Epithemia turgida	(Ehrenberg) Kützing
Eunotia bilunaris	(Ehrenberg) Mills
Fragilaria arcus	*(Ehrenberg) Cleve*
Fragilaria capucina	Desmazières
Fragilaria construens	(Ehrenberg) Grunow
Fragilaria pinnata	Ehrenberg
Fragilaria virescens	Ralfs
Frustilia vulgaris	(Thwaites) De Toni
Gomphonema angustatum	Kützing
Gomphonema clavatum	Ehrenberg
Gomphonema clevei	Fricke
Gomphonema parvulum	Kützing
Gomphonema type D	
Melosira varians	Agardh
Meridion circulare	(Greville) Agardh
Navicula atomus	(Kützing) Grunow
Navicula cryptotenella	Lange-Bertalot
Navicula gallica	(W. Smith) Lagerstedt
Navicula minima	Grunow
Navicula placentula	(Ehrenberg) Grunow
Navicula tripunctata	(O.F.Müller) Bory de Saint-Vincent
Navicula variostriata	Krasske
Navicula viridula	(Kützing) Kützing
Nitzschia aequorea	*Hustedt*
Nitzschia dissipata	(Kützing) Grunow
Nitzschia fonticola	Grunow (Grunow)

Nitzschia inconspicua	Grunow
Nitzschia palea	(Kützing) W. Smith
Nitzschia paleacea	Grunow
Pinnularia spp.	
Rhoicosphenia abbreviata	(Agardh) Lange-Bertalot
Rhopalodia gibba	(Ehrenberg) O. Müller
Surirella angusta	Kützing
Synedra ulna	Ehrenberg
